# Multiple Thyrotropin β-Subunit and Thyrotropin Receptor-Related Genes Arose during Vertebrate Evolution

**DOI:** 10.1371/journal.pone.0111361

**Published:** 2014-11-11

**Authors:** Gersende Maugars, Sylvie Dufour, Joëlle Cohen-Tannoudji, Bruno Quérat

**Affiliations:** 1 Muséum National d'Histoire Naturelle, Sorbonne Universités, Biology of Aquatic Organisms and Ecosystems (BOREA), Paris, France, Université Pierre et Marie Curie, Paris, France, Université Caen Basse Normandie, Caen, France, Unité Mixte de Recherche (UMR) 7208 Centre National de la Recherche Scientifique (CNRS), Paris, France, Institut de Recherche pour le Développement (IRD) 207, Paris, France; 2 Université Paris Diderot, Sorbonne Paris Cité, Biologie Fonctionnelle et Adaptative (BFA), Paris, France, UMR CNRS 8251, Paris, France, INSERM U1133 Physiologie de l'axe gonadotrope, Paris, France; University of Lausanne, Switzerland

## Abstract

Thyroid-stimulating hormone (TSH) is composed of a specific β subunit and an α subunit that is shared with the two pituitary gonadotropins. The three β subunits derive from a common ancestral gene through two genome duplications (1R and 2R) that took place before the radiation of vertebrates. Analysis of genomic data from phylogenetically relevant species allowed us to identify an additional *Tshβ* subunit-related gene that was generated through 2R. This gene, named *Tshβ2*, present in cartilaginous fish, little skate and elephant shark, and in early lobe-finned fish, coelacanth and lungfish, was lost in ray-finned fish and tetrapods. The absence of a second type of TSH receptor (*Tshr*) gene in these species suggests that both TSHs act through the same receptor. A novel *Tshβ* sister gene, named *Tshβ3*, was generated through the third genomic duplication (3R) that occurred early in the teleost lineage. *Tshβ3* is present in most teleost groups but was lostin tedraodontiforms. The 3R also generated a second *Tshr*, named *Tshrb*. Interestingly, the new *Tshrb* was translocated from its original chromosomic position after the emergence of eels and was then maintained in its new position. *Tshrb* was lost in tetraodontiforms and in ostariophysians including zebrafish although the latter species have two TSHs, suggesting that TSHRb may be dispensable. The tissue distribution of duplicated *Tshβs* and *Tshrs* was studied in the European eel. The endocrine thyrotropic function in the eel would be essentially mediated by the classical *Tshβ* and *Tshra*, which are mainly expressed in the pituitary and thyroid, respectively. *Tshβ3* and *Tshrb* showed a similar distribution pattern in the brain, pituitary, ovary and adipose tissue, suggesting a possible paracrine/autocrine mode of action in these non-thyroidal tissues. Further studies will be needed to determine the binding specificity of the two receptors and how these two TSH systems are interrelated.

## Introduction

Thyroid-Stimulating Hormone (TSH) is a pituitary glycoprotein hormone responsible for the activation of the thyroid gland, playing a key role in the control of development and metabolism in mammals and other vertebrates [1]. TSH is also responsible for triggering specific developmental processes such as larval metamorphosis in amphibians [2], [3], as well as larval and secondary metamorphoses in some teleost species [4]–[8]. In addition, TSH may participate in the modulation of various functions for example in the immune or reproductive systems, *via* pleiotropic effects and multiple target tissues of thyroid hormones [9]–[12].

The vertebrate pituitary glycoprotein hormones, TSH and the two gonadotropins, luteinizing hormone (LH) and follicle-stimulating hormone (FSH) are heterodimers composed of a common α subunit, and a β subunit that confers hormonal specificity [13]. It has recently been demonstrated [14] that the three glycoprotein hormone β (GPHβ) subunits were generated by successive duplications starting from an ancestral glycoprotein hormone β subunit gene (*ancGphβ*) through two rounds of genomic duplications (1R and 2R) that occurred early in the evolution of vertebrates [15]. During 1R the original *ancGphβ* duplicated into two paralogous genes, one of which became the evolutionary precursor of the gonadotropin β subunit genes (*preGthβ*) and the other, the precursor of the *Tshβ* subunit gene (*preTshβ*). *Lhβ* and *Fshβ* were generated next by the duplication of *preGthβ* during 2R. *Tshβ* derived from *preTshβ* but the presence of a 2R-derived *Tshβ* subunit sister gene has never been demonstrated [14]. Analysis of the glycoprotein hormone related gene repertoire of the elephant shark (*Callorhinchus milii*) interestingly revealed the presence of two copies of *Tshβ* subunit related genes. Whether they resulted from a specific, local duplication of the *Tshβ* gene or from the conservation in cartilaginous fish of the *Tshβ* subunit sister gene derived from the 2R, could not be determined at that time [14].

The scenario appears rather similar in extant teleosts [16] despite the specific genomic duplication (3R) that took place in this lineage [17]. A *Tshβ* sister gene was however identified in some teleost genomes, that was shown to be derived from the 3R [14], [18].

The glycoprotein hormones exert their action by interacting with specific and evolutionarily related G protein-coupled receptors. The glycoprotein hormone receptors (GPHR) are characterized by a large extracellular hormone-binding domain composed of a leucine rich domain connected to a seven-transmembrane domain by a hinge region [19]. If a second TSH related hormone is present in some species, it seems logical to assume that it acts through a novel receptor, as suggested by the recent characterization of a second TSHR-like gene in some teleost species [20], [21]. However, it was not clearly demonstrated whether this second TSHR was generated at the 3R or by a specific duplication early in the teleost lineage.

Whether additional *Tshβ* subunit genes and *Tshr* genes were derived from 2R and 3R were questions we addressed in this study. We took advantage of the recently released genomic data from several species that have a phylogenetically relevant position among vertebrates: two representatives of cartilaginous fish (chondrichthyes), a group that preceded the divergence of ray-finned fish (actinopterygies) and lobe-finned fish (sarcopterygies), the elephant shark, an holocephalan for which a new version of the genomic assembly was recently released [22] and the little skate (*Leucoraja erinacea*), an elasmobranch; the spotted gar (*Lepisosteus oculatus*) a ray-finned fish representative that took root before the teleost radiation and the 3R [23] and the coelacanth (*Latimeria chalumnae*), a lobe-finned fish that appeared just prior to lungfish [24], the lungfish group being the sister group of tetrapods [25]. The genomes of a number of teleost fish species including the eels (*Anguilla anguilla* and *A. japonica*), representatives of the basal group of Elopomorphs [26]–[29] also recently released, were searched for the 3R generated *Tshβ* related subunit and for a *Tshr* related gene. The tissue distribution of the two *Tshβ* and the two *Tsh receptors* was analysed in the European eel.

## Materials and Methods

All aspects of animal care and experimentation were in accordance with the Ethic committee of the Museum National d'Histoire Naturelle and approved by the Institutional Animal Care and Use Committee of the Animal Protection and Health, Veterinary Services Direction, Paris, France.

### Identification of vertebrate *Tshβ* and *Tshr* sequences

Blast analyses [30] were performed on-line using protein as query (tBlastn) on NCBI (http://blast.ncbi.nlm.nih.gov/Blast.cgi), Ensembl (http://www.ensembl.org/Multi/blastview), DDBJ (http://blast.ddbj.nig.ac.jp/), as well as on web sites for little skate (Skatebase: http://skatebase.org/skateBLAST and elephant shark (http://esharkgenome.imcb.a-star.edu.sg/blast/). Eel sequences were identified from European and Japanese eel genomes available on the website Eel Genome of ZF-Genomics (http://www.zfgenomics.org/sub/eel) in addition to the assembly available in NCBI, using the CLC BIO software (Qiagen, Denmark).

Protein sequences were predicted from retrieved genomic or Expressed Sequence Tag (EST) sequences by using consensus splice donor and acceptor site and by sequence identity comparison with related *Tshβ* or *Tshr* genes (Table S1 and Table S2).

The signal peptide cleavage site was determined using SignalP (http://www.cbs.dtu.dk/services/SignalP/). Receptor transmembrane domains were predicted using TOPCONS (http://topcons.cbr.su.se/).

### Phylogenetic and syntenic analyses

Alignments were fitted manually using Se-AL editor (http://tree.bio.ed.ac.uk/software/seal/). The phylogenetic reconstructions were performed on-line by using a maximum likelihood method with PhyMyL 3.0 software [31] on the website file (http://www.phylogeny.fr/) with HKY85 as substitution model for TSHβ-related nucleotide sequences and WAG for TSHR-related amino-acid sequences and default settings for the other parameters. The robustness of the reconstruction was estimated by the aLRT score and/or by bootstrapping over 500 replicates. Nucleotide sequences of the entire coding region (including signal peptide) were used for *Tshβ* subunits with truncation in the 3′ end of the longest sequences. The amino acid sequences were used for the TSHR tree reconstruction.

Mapping the genomic neighborhoods of *Tshβ* and *Tshr* genes were performed with region overview on Ensembl, NCBI and EBI genome browsers and for the Elephant shark genome on the specific Ensembl website (http://ensembl.fugu-sg.org/index.html).

Flanking genes of duplicated *Tshβ* and *Tshr* were identified and annotated in the eel from the eel genome databases, using CLC BIO software.

### Tissue distribution of *Tshβ* and *Tshr* transcripts in the eel

Tissue distribution analysis was performed on RNA samples previously prepared from female silver migrating eels caught in the River Loire, France [32]. Total RNA extracted from pituitary, thyroid follicles, olfactory bulb, mesencephalon and diencephalon, telencephalon, cerebellum, medulla oblongata, eyes, liver, intestine, muscle, adipose tissue, gills, and ovary were used. Reverse transcription was performed as previously described [32].

Primers for quantitative real-time PCR (qPCR) for European eel *Tshβ* (Table S3) were previously reported [32]. Eel specific primer sets for *Tshβ3*, *Tshra* and *Tshrb* were designed using Primer3 [33], [34] spanning intron sequences. The specificity of the primer sets was controlled by sequencing PCR product. Moreover, in each case, we checked that the isolated cDNAs of one of the duplicated genes could not be amplified by the primer set corresponding to the other duplicated gene.

Messenger RNA was quantified on LightCycler using the LightCycler FastStart Master plus Sybr green I kit (Roche, Mannhein, Germany) as recommended by the manufacturer. The final primer concentration used was 500 nM. Each sample was run in duplicate using a 1/5 cDNA dilution. The PCR conditions were 95°C for 10 min followed by 50 cycles at 95°C for 5 sec, 60°C for 10 sec and 72°C for 10 sec. The specificity of amplified product was checked by melting curve analysis after the amplification reactions. Relative transcript abundance was calculated from standard curves prepared from pituitaries and thyroid follicles cDNA using LightCycler software. Transcript levels were normalized using total tissue RNA content as previously described in [35].

## Results and Discussion

### A 2R-generated *Tshβ* subunit related gene conserved in cartilaginous and in basal lobe-finned fish

**Figure 1:**
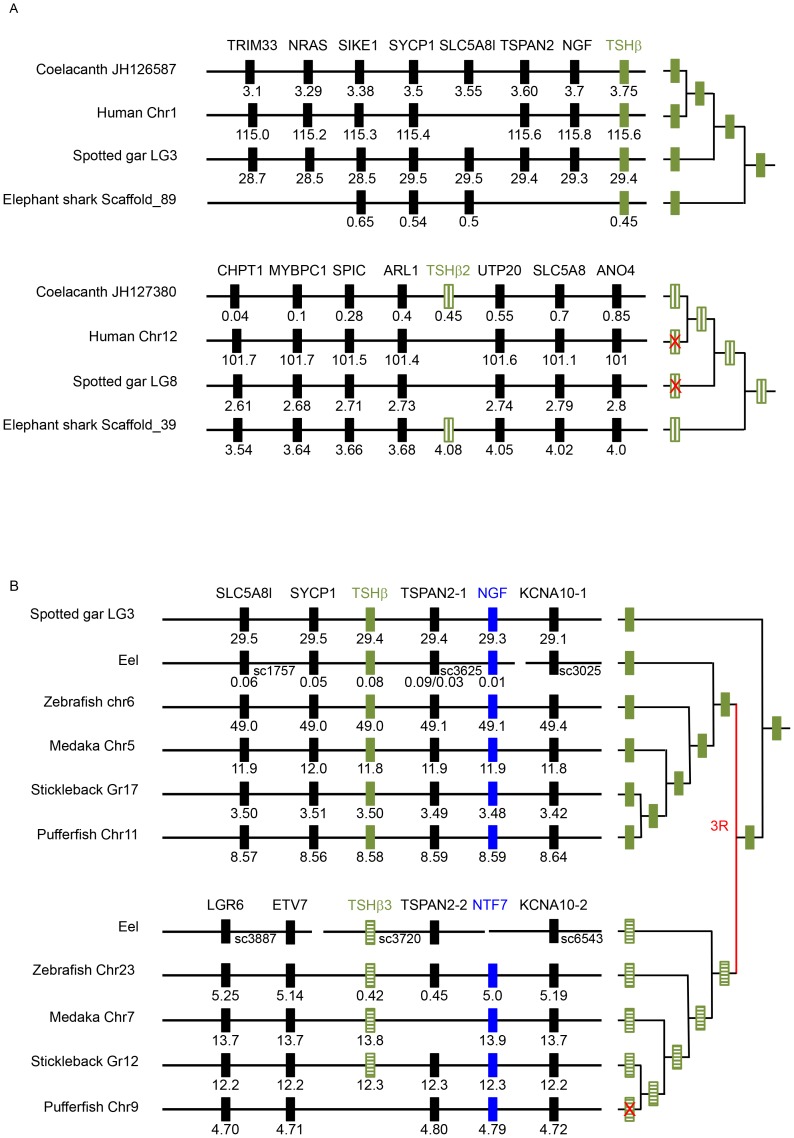
Syntenic analysis of *TSHβ* related gene regions. Genomic regions of *Tshβ vs Tshβ*2 genes (Panel A) and *Tshβ vs Tshβ3* genes (Panel B) were analysed in representative species (chromosome number or linkage group references are attached to the species name) by using the region overview on the Ensembl genome browser or by blast analysis on the eel draft genome (see Fig. S2A for details). The phylogenetic relationships between the representative species are summarized on the right panel. The 3R symbolizes the teleost-specific genome duplication. Genes are named according to the Ensembl nomenclature (Table S4). Gene positions are given (in Mega base) below the symbolized genes.

Two related *Tshβ* subunit genes were characterized from the coelacanth genome (Fig. 1A). One of them was in the same genomic region as the known “classical” vertebrate *Tshβ* subunit gene. A second gene was located on a genomic fragment that encodes several genes among which three (*Mycbpc1*, *Spic* and *Ano4*) were demonstrated to belong to the fourth paralogous group of genes derived from the duplicated *ancGphβ* genomic region (see additional file 9 in [14]). This fourth glycoprotein hormone β subunit gene then clearly represents the *Tshβ* sister gene derived from the 2R and was named *Tshβ2*.

The genes for the two *Tshβ* subunit cDNAs (HQ174785 and HQ174784) previously characterized in elephant shark from a pituitary library [14] were present and complete in the new version of the genome, on scaffold_89 and scaffold_39, respectively. The former was flanked by genes belonging to the “classical” *Tshβ* subunit paralogous gene set (Fig. 1A). This was the one unfortunately named *Tshβ2* when first characterized [14]. The other *Tshβ* subunit gene on scaffold_39 was co-syntenic with genes located in the same genomic region as the coelacanth *Tshβ2* (Fig. 1A). The hypothesis formulated at the time that one of these genes may be the *Tshβ* sister gene issued from the 2R is thus confirmed.

**Figure 2:**
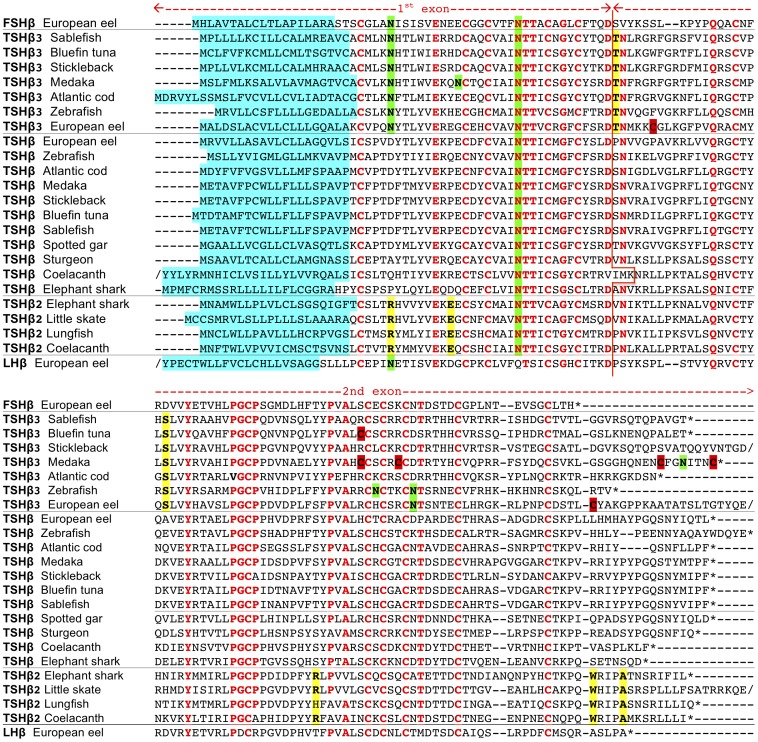
TSH*β*-related sequence alignment. Amino acid sequence alignment of representatives of the three groups of TSHβ subunits. Eel LHβ and FSHβ sequences are given for comparison. The predicted signal peptide is highlighted in blue. The exon splicing site is indicated by the red vertical bar. Overall conserved amino acids are in bold red. Specific positions in TSHβ2 or -β3 sequences relative to TSHβ are highlighted in yellow. Potential glycosylation sites are highlighted in green. Additional, non-conserved cysteine residues are highlighted in red. Sequences might be truncated in the signal peptide or the carboxy-terminal end for convenience. Full-length sequences are presented in Fig. S1 and references are given in Table S1.

The skate genome is not fully assembled yet and most of the genes of interest were fragmented into as many contigs as coding exons. The first and second exons of the *Tshβ* subunit related gene were identified and tentatively linked (Fig. 2).

Only one *Tshβ* subunit gene was found in the spotted gar genome. It was located on the same genomic region as the classical *Tshβ* subunit gene (Fig. 1A).

### A 3R-generated *Tshβ* subunit-related gene conserved in most teleosts

The classical *Tshβ* and a second *Tshβ* subunit in teleosts were confirmed in a number of representatives from basal elopomorphs like the eel to the acanthomorphs (stickleback, tilapia, tuna, sablefish) through ostariophysian species (Mexican tetra, zebrafish) (Fig. 2, Fig. S1 and Table S1). The first exon of a second *Tshβ* was also identified in the Atlantic salmon (*Salmo salar*) (Fig. S1, Table S1) suggesting that salmonids also have this second form of TSH. This was not the case in tetraodon and fugu species where only the classical and already characterized *Tshβ* subunit genes could be found in the complete genome. Synteny analysis shows that the additional *Tshβ* subunit is located in a conserved genomic region (Fig. 1B), close to the 3R issued duplicated form of *Ngf*, *Ntf7* [36]. This additional Tshβ gene was named *Tshβ3* with reference to the 3R.

### *Tshβ* subunit sequences part into three monophyletic groups

**Figure 3:**
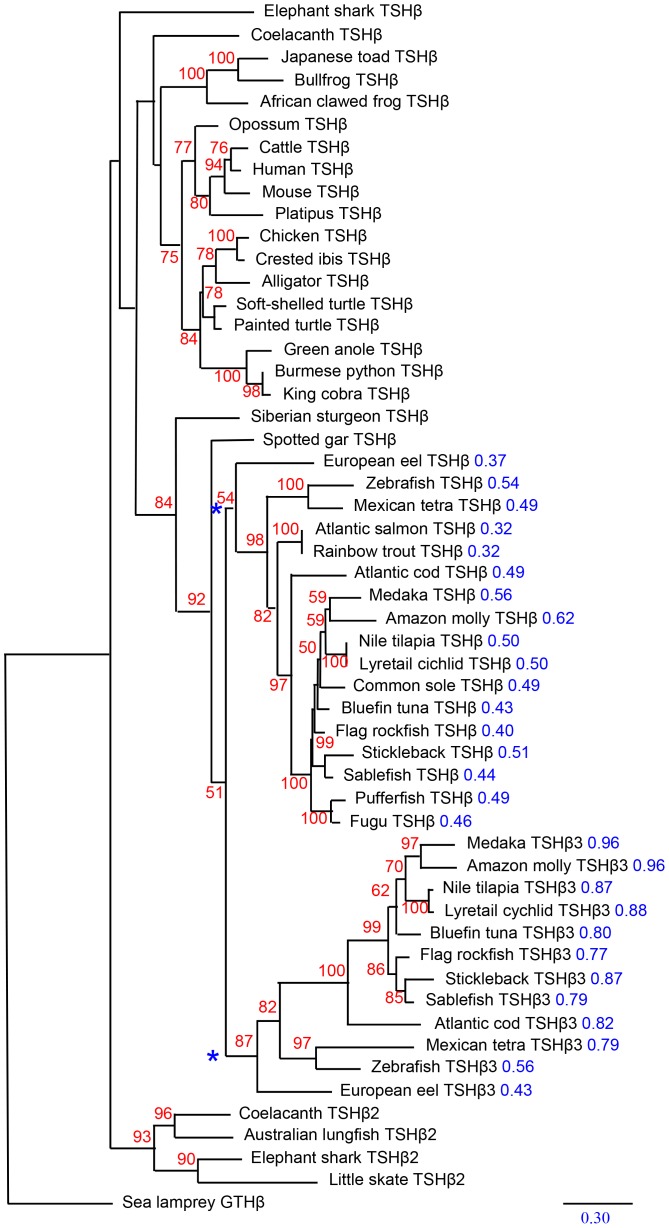
Phylogenetic tree of TSHβ related nucleotide sequences. Phylogram of maximum likelihood relationships between *Tshβ* coding sequences of representative species. The bootstrap values over 500 replicates (in %) are given next to each node in red (only the values above 50% are given). Cumulated distance values (from the node marked with a blue asterisk) are given in blue next to the species name for comparison of the estimated relative rate of evolution of teleost TSHβ and TSHβ3 sequences (see Fig. S4 for the regression curve). *Tshβ* gene references are given in Table S1.

*Tshβ* subunit sequences of vertebrate representatives were aligned for a phylogenetic analysis. As expected from the synteny analysis, a monophyletic group emerged that clusters the coelacanth *Tshβ2* together with the elephant shark *Tshβ*-related subunit HQ174784 (Fig. 3), the protein deduced from the assembled *Tshβ* exons from the skate and the known *Tshβ* from the Australian lungfish [37]. The robustness of the monophyletic group that constitutes a sister group to all other *Tshβ* subunits was strongly supported by a bootstrap value of 93% in 500 replicates. These *Tshβ* genes were named *Tshβ*2. The other coelacanth and elephant shark *Tshβ* genes were included into the “classical” *Tshβ* cluster at positions compatible with their phylogenetic relationships

Although two *Tshβ* related genes were identified in elephant shark, only one was tentatively characterized in the skate. It branches out with the coelacanth *Tshβ2*. The classical *Tshβ* subunit sequence was not found. One possibility is that only one *Tshβ* subunit was conserved in skate or in holocephals. More likely, however, since *Ngf* was also absent from the genomic data, the entire locus may have been missed in the sequencing process.

Teleost *Tshβ* sequences were divided into two monophyletic groups. This is in agreement with the syntenic analysis and supports the hypothesis that they result from the third genomic duplication (3R) that took place early in the radiation of teleosts. The *Tshβ3* sequence branch length from this phylogenetic tree was 1.6 longer in average (Fig. S4) than for the classical *Tshβ* sequences indicating that they evolved more rapidly.

The spotted gar belongs to a group that emerged before the radiation of teleosts and its specific genome duplication. It logically lacks the *Tshβ3* gene. It also lacks the *Tshβ2* gene although the genomic region, where it should be located, is well conserved (Fig. 1A) discarding a possible problem with the sequencing data. It then seems that the *Tshβ2* gene was lost at least twice, in the lobe-finned fish lineage before the radiation of tetrapods and early in the ray-finned fish lineage. It must be of significance that teleosts specifically retained the newly generated *Tshβ3* gene when they lost the other 3R-generated *Gphβ* gene duplicates [38].

### TSHβ2 and TSHβ3 sequences present specific signatures

Both TSHβ2 and TSHβ3 retained most structural features shared by all types of glycoprotein hormone β subunits like the cysteine residues and many other amino acids that are conserved in position (Fig. 2) indicating that these sequences are subject to functional constraints. It is then most likely that they are able to associate to an α subunit and form an active heterodimer. TSHβ2 and TSHβ3 are predicted to be cleaved from the signal peptide at roughly the same position as in the classical TSHβ, one or two amino acids before the first conserved cysteine residue (Fig. 2). Sequence alignment indicates that five amino acid positions are well conserved in the TSHβ2 group that are different to or variable in the classical TSHβ subunit sequences. Two are located within the first exon and 3 within the second. The amino acid composition from the two associated skate exonic sequences are well in agreement with their assembly into a unique gene belonging to the *Tshβ2* group. TSHβ2 and TSHβ subunits share the two additional amino acid residues between the cysteines 5 (the last encoded by the 1^st^ exon) and 6 (20 amino acids apart) as compared to LHβ and FSHβ subunits [39]. By comparison, in the tunicate ciona GPB5, like in the gonadotropin β subunits, the homologous cysteine residues are 18 amino acids apart [40], [41]. Given tunicates are the closest relatives to vertebrates [42]and since *ancGphβ* was generated by a duplication of *Gpb5* just prior to the emergence of vertebrates[14], it is likely in the ancGPHβ subunit precursor, the cysteines were also 18 amino acids apart. Thus, the *preTshβ* evolutionary precursor likely acquired these two codon insertions at the time of the 1R. It can be inferred from the aligned sequences that the insertion/deletion event was not generated at the splice site (Fig. 2). In the coelacanth TSHβ sequences however, the splice site is shifted twelve nucleotides towards the 3′ end.

TSHβ3 sequences display particular signatures (Fig. 2 and Fig. S1). The most significant is that TSHβ3-type subunits harbor two potential N-linked glycosylation sites. The glycosylation pattern of vertebrate glycoprotein hormone β subunits is usually well conserved with two sites in FSHβ and most likely in the ancestral β subunit, whereas LHβ have kept one site (the one towards the N-terminal), and TSHβ the other. The *Tshβ* subunit precursor gene that was duplicated during the 3R encoded a subunit with only one glycosylation site found, at the second position, as for all classical TSHβ subunits. The additional site was then *de novo* created by mutation of a well-conserved aspartic acid (D) to an asparagine (N) at the first position, two amino acids upstream of a conserved threonine. Since glycosylation sites are more likely to be created by generating a serine or threonine residue downstream of an existing asparagine [43], there may be some kind of structural constraints for this glycosylation site to be re-created precisely at this position. Similar constraints should have applied for human LHβ subunit, also characterized by a glycosylation site that is, conversely, switched from the first position to the second, TSH-type position. Other scattered potential glycosylation sites are observed in some TSHβ3 sequences (Fig. 2). Another feature of these TSHβ3 sequences is the presence of additional cysteine residues, up to maximum of four in the medaka sequence. Whether these cysteine residues are involved in generating intra- or inter-subunit disulfide bonds remains to be determined.

### A single receptor for TSH and TSH2 in cartilaginous fish and basal lobe-finned fish

Only one receptor gene was identified in the coelacanth and elephant shark genomes, although they have two TSHβ subunits. It is thus likely that the additional TSH2 made up of TSHβ2 and the common α subunit, would act through binding to the same TSH receptor, as the classical TSH. Such a redundancy might have led to the loss of the second TSH in tetrapods and in ray-finned fish. The lungfish *Tshβ2* subunit cDNA [37] as well as the two *Tshβ* cDNAs from the elephant shark [14] were cloned from pituitary libraries. In situ hybridization studies will be needed to determine whether they are produced by the same cells. Examination of tissue distribution could also reveal whether they are expressed in non-pituitary tissues.

### One or two potential receptors for TSH and TSH3 in teleosts

**Figure 4:**
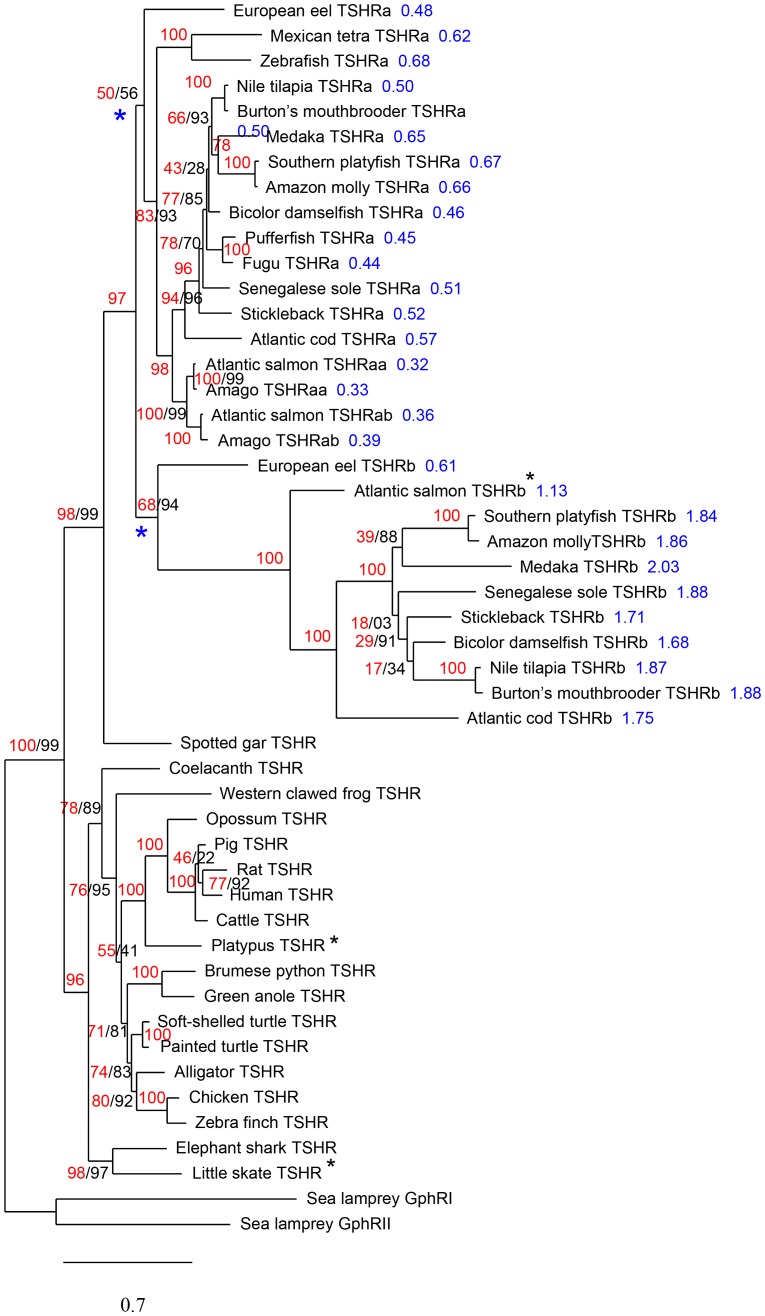
Phylogenetic tree of TSHR-related amino acid sequences. Phylogram of maximum likelihood relationships between TSHR amino acid sequences of representative species. The bootstrap values (in %) are given next to each node in red and the SH-like aLRT scores are given in black (when different from the boostrap value). Cumulated distance values (from the node marked with a blue asterisk) are given in blue next to the species name for comparison of the estimated relative rate of evolution of teleost TSHRa and TSHRb sequences (see Fig. S4 for the regression curve). *Tshr* gene references are given in Table S2. Black asterisks indicate partial sequences.

**Figure 5:**
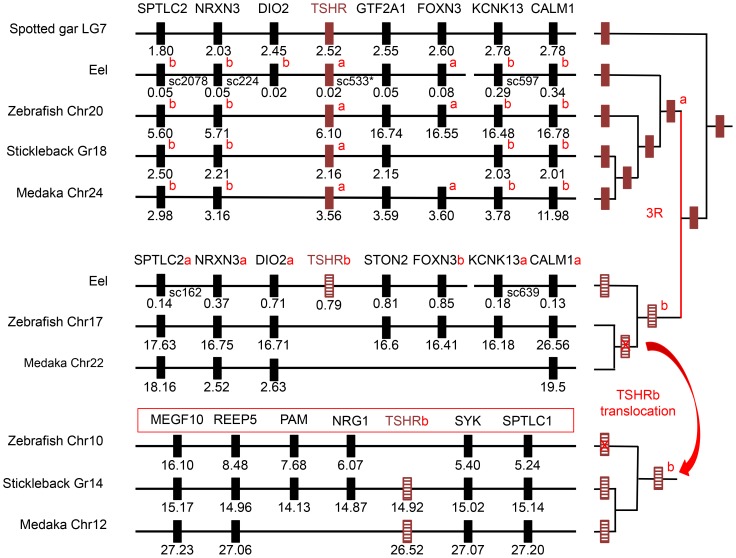
Syntenic analysis of TSHR-related genomic region. Genomic region flanking *Tshr*-related genes were analysed in representative species (chromosome number is attached to the species name) by using the region overview on the Ensembl genome browser or by blast analysis on the eel assembled genome (see Fig. S2B for details). The phylogenetic relationships between the represented species are summarized on the right panel. *Tshrb* was translocated sometime between the emergence of eel and stickleback lineages. Genes are named according to the Ensembl nomenclature (Table S4). Gene positions are given (in Mega base) below the symbolized genes.

Two TSH receptor sequences were identified in most teleost groups (Fig. S3). Phylogenetic analysis (Fig. 4) showed that teleost TSHR are divided into two monophyletic groups, each one with one eel TSHR-type branching at a basal position. In contrast, only one TSHR was found in the spotted gar, confirming that the duplication of *Tshr* occurred early in the teleost radiation [20], [21]. Synteny analysis revealed that one of the teleost duplicated receptors is conserved in the same chromosomal region as before the duplication event (Fig. 5). This is the *Tshra*-type receptor as previously named [21]. The eel *Tshrb* is maintained in a similar genomic region as *Tshra*, in agreement with it resulting from the 3R (strict double conserved synteny). In contrast, in more derived teleost species such as cod, medaka and stickleback, *Tshrb* was found in a new genomic region (Fig. 5 and Table S4). This indicates that *Tshrb* was translocated some time after the emergence of Elopomorphs to another environment where its new location was stabilised. This change in the genomic environment of the *Tshrb* gene may alter the control of its expression, notably through epigenetic modifications and thus its response to environmental factors [44], [45].

**Figure 6:**
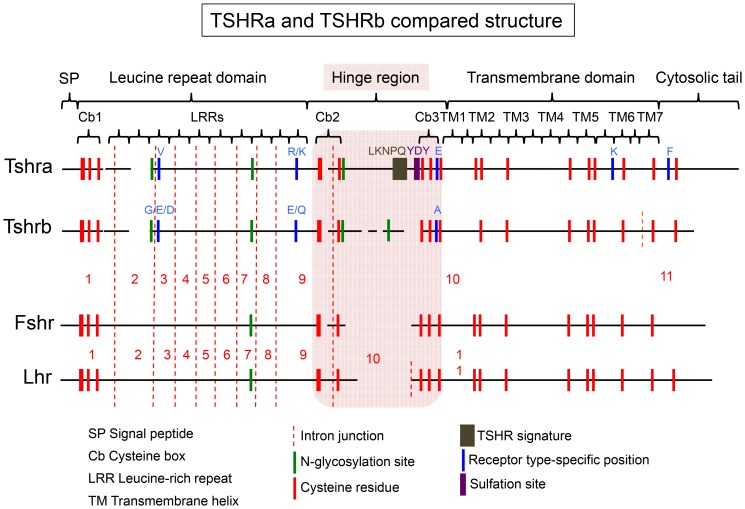
Schematic representation of TSHRa and TSHRb sequence features. Representation of the two types of teleost TSHR. LHR and FSHR are given for comparison. The exons splicing sites are indicated by a red dotted vertical bar. Both TSHR have conserved the typical architecture of the glycoprotein hormone receptor composed of a long extracellular domain comprising, from N- to C-terminal, a signal peptide (SP); a leucine-rich domain formed by successive leucine-rich repeat (LRR); a hinge region (in pink); a seven-transmembrane domain composed of seven helical regions (TM) connected by intracellular and extracellular loops alternatively and a cytosolic tail. Cysteine boxes at the N-terminal of the leucine rich domain and flanking the hinge are indicated (cb). Cysteine residues are in red, N-glycosylation sites in green. TSH-specific motif is in brown and sulfation site (YDY) in purple. Specific amino acids are labelled in blue.

Like vertebrate *Tshr*, teleost *Tshra* is encoded by 10 exons. Nine of them encode the leucine-rich repeat (LRR) domain and, the large 10^th^ exon encodes the transmembrane domain together with the carboxy-terminal cytosolic tail (Fig 6 and Fig. S3). This structure is conserved in eel *Tshrb*. In contrast, the domains encoded by the 10^th^ exon appeared to be split into two exons in *Tshrb* from more derived teleosts (Fig. S3). Furthermore, the intracellular domain which usually exhibits signalization and internalization properties appeared to be shorter in TSHRb in these teleosts. However, alternative or additional exons could have been missed in our tentative sequencepredictions. Cloning of full length *Tshrb* cDNA in these teleost species could validate shortening of the cytosolic tail.

In Atlantic salmon, three *Tshr* sequences could be reconstructed from genomic data (Table S2). Phylogenetic analysis (Fig. 4) shows that two of them branch out together with the two characterized Amago receptors [46] and are of the a-type as previously shown [21]. These two a-type receptor genes likely result from the salmonid-specific genome tetraploidization event (4R) [47]. A third, partial Atlantic salmon *Tshr* sequence identified here appears to be of the b-type indicating that salmonids are likely to have both types of TSHR.

A single *Tshr* (*Tshra*) is present in the genome of tetraodontiformes (e.g. fugu, pufferfish). The loss of the duplicated *Tshrb* in this teleost group is in agreement with the fact that they possess only one TSH. In contrast, *Tshrb* was also absent in ostariophysians (zebrafish, Mexican tetra), while they possess two potential TSHs. This indicates at least two independent losses of *Tshrb* during the radiation of teleosts. As with coelacanth or elephant shark TSH and TSH2, ostariophysian TSH and TSH3 might act through a unique TSHR receptor.

### TSHRa and -b sequences present specific signatures

The TSHR sequence alignment showed high conservation features throughout the vertebrate evolution (Fig 6, Fig. S3). As compared with FSHR and LHR sequences, both TSHR types present a specific long hinge region, delimited by two conserved cysteine boxes (cb2 and cb3) connecting the extracellular domain to the transmembrane domain. The fact that most of these TSHR structural features were maintained in TSHRb indicates that it is subjected to similar functional constraints.

In both types of TSHR, two potential glycosylation sites were conserved, one close to the LRR2 region (N88 of the alignment in Fig. S3) and the other at the end of LRR6 (N210), the latter being common to all glycoprotein hormone receptor types. The N-glycosylation site at the end of the cysteine box 2 (N324) was also conserved in both types of TSHR (except for the medaka TSHRb). The N-glycosylation site found in tetrapod sequences at the end of the LRR5 (N188) is conserved in lobe-finned fish and cartilaginous fish but is absent in ray-finned fish. Except in the eel, teleost TSHRb have additional potential N-glycosylation sites within the hinge region. They also differ from all other TSHR by lacking a conserved negative charge at position 262 at the start of the LRR9. A low amino acid conservation was observed within the hinge region for the TSHRb compared with the other TSHR (Fig. S3). In addition, one key cysteine of the cysteine-box 3 that was demonstrated to be involved in the disulfide bonding that gives its conformation to the hinge region is lost in cod. The THSRb hinge region lacks the common TSHR signature LKNPQ. The highly conserved tyrosine sulfation motif site [Y-(DE)-Y] within the hinge region involved in hormone recognition and signal transduction [48] that is still present in the eel is not conserved in more derived teleosts. In addition, the conserved glutamic acid (E) at position 446, which is a key determinant for the activation of the receptor [49], was switched to an alanine. Whether these differences in structure impact on binding specificity and on the signaling pathway will have to be determined.

TSHRb diverged at many points after the emergence of the eel as reflected by longer phylogenetic branch lengths (3.1 times that of TSHRa; cf Fig. 4 and Fig. S4), demonstrating an accelerated rate of evolution. Such a change in evolution rate is likely related to the duplication event with one gene keeping its original features (the type-a receptor) allowing the other to acquire new specificities in spatial or temporal control of its expression and in binding characteristics of its encoded protein [15], [50]. Studies of functionality of the duplicated TSH and TSHR should be addressed in the future by developing recombinant hormone and receptors for various teleost species. Such investigations are required to characterize the binding selectivity of the two TSHR and to determine whether they activate the same signaling pathway.

### A dual TSH system in the European eel

**Figure 7:**
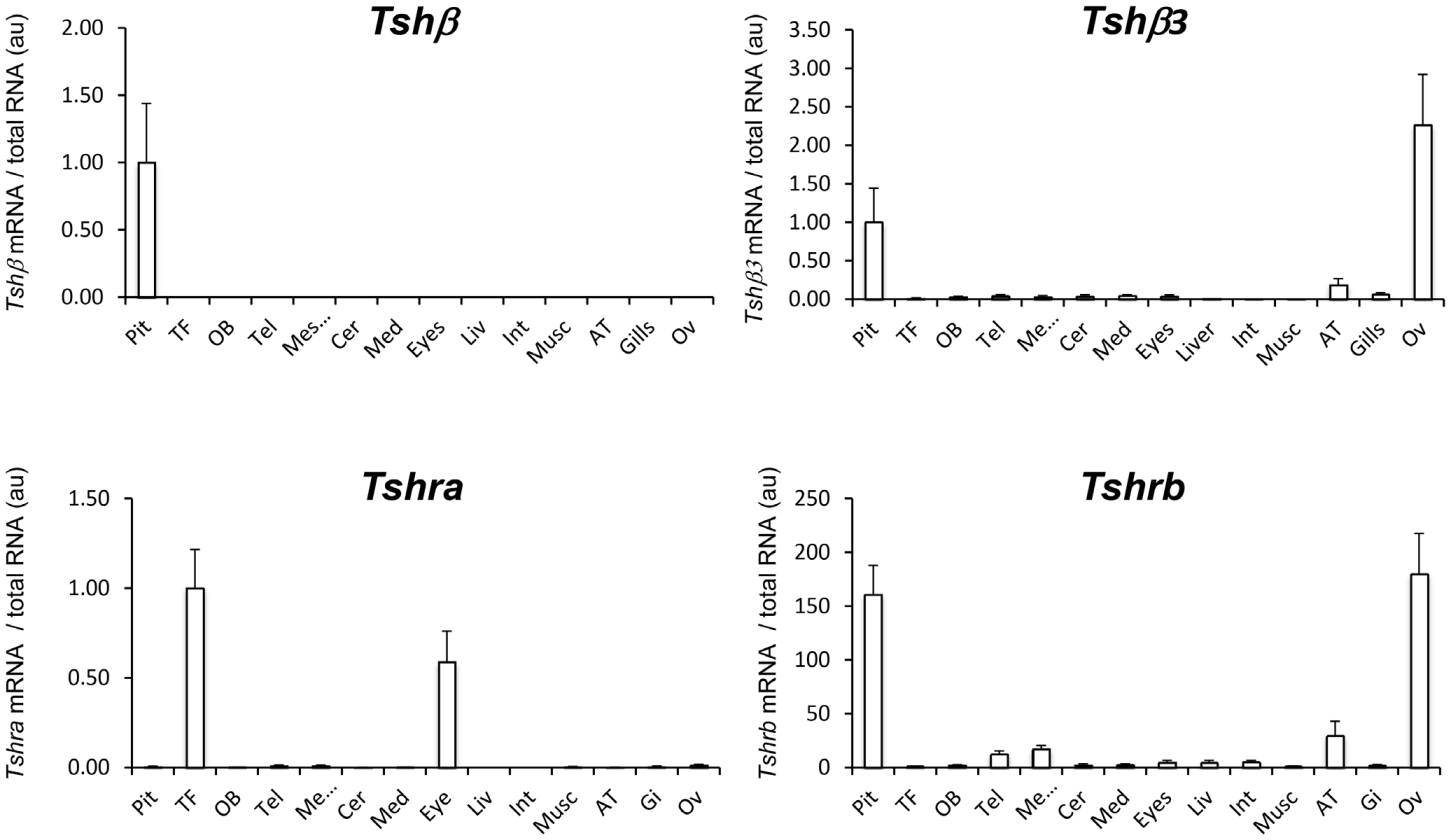
Tissue distribution profile of *Tshβ*, *Tshβ3*, *Tshra* and *Tshrb* mRNA in the eel. Tissue distribution was analysed by RT-qPCR on RNA extracted from various tissues in the immature female European eel. Owing to the different nature of the tissues, transcript levels were normalized using total tissue RNA content: pituitary (Pit), thyroid follicles (TF), olfactory bulb (OB), mes-/diencephalon (Mes/), telencephalon (Tel), cerebellum (Cer), medulla oblongata (Med), eyes, liver, intestine (Int), muscle (Musc), adipose tissue (AT), gills, and ovary (Ov). Transcript levels of *Tshβ* subunit and of *Tshr* were normalized to the level in the pituitary and in the thyroid follicles, respectively and are expressed as arbitrary units. Results are represented as mean values ± SEM (n = 8).

The tissue distribution analysis by qPCR of the two duplicated *Tshβ* transcripts in the immature female eel showed that the classical *Tshβ* subunit was exclusively expressed in the pituitary (Fig. 7). A low expression of *Tshβ3* could also be detected in the pituitary. Further *in situ* hybridization studies will be needed to pin-point if both duplicated *Tshβ* are expressed by the same pituitary cells. In addition, *Tshβ3* was highly expressed in the ovary and was detected in adipose tissue, gills, brain structures and eye. This is the first report of a comparative tissue distribution of the two *Tshβ* subunits in teleosts. It clearly shows that the two β subunits have a differential tissue expression, which may represent one of the evolutionary drives leading to the conservation of the duplicated hormone. In stickleback, *Tshβ* and *Tshβ3* expression was compared only in the pituitary where both are expressed but *Tshβ3* showed differential transcriptional regulation according to the ecotype [18]. Unlike in the eel, the classical *Tshβ* subunit was shown to be expressed not only in the pituitary but also in the gonads of the fathead minnow [51] and the grouper [52]. EST data mining indicates that *Tshβ* transcripts are expressed in the brain of several teleost species (zebrafish - EH470445.1 and goldfish - DY231942; tilapia - GR616157.1; medaka - DK0206541) unlike the eel. In particular, *Tshβ* transcripts are found in the *saccus vasculosus*, at the base of the hypothalamus in salmon and may be involved as a photoperiodic signal transducer [53]. It is too early to draw conclusions about the absence of significant expression of the classical TSH in other tissues than the pituitary in the eel. Whether it is linked to its basal phylogenetic position relative to the duplication event, the physiological stage of the silver eel, or species specificity in the respective functions of the two TSHs will have to be further explored.

*Tshβ3* was identified among transcripts isolated from liver in the adult Antarctic toothfish (FE210400.1) and from the ovary in the yellow perch (GO658547.1) indicating that the expression of this gene in these species as well as in the eel, is not restricted to the pituitary gland.

In the eel, as with the two *Tshβ* subunits, the duplicated *Tshr* showed a differential tissue distribution pattern. The thyroid follicles showed a high expression of *Tshra* while a low level of *Tshrb* transcript could be detected (Fig.7). This suggests that Tshra would mediate the classically described thyrotropic endocrine function of TSH. This is in agreement with the conserved synteny and sequence of teleost *Tshra* as compared with tetrapod *Tshr*. Together with the major expression of the classical *Tshβ* in the eel pituitary, this allowed us to infer that endocrine control of thyroid function via the classic TSH produced by the pituitary and classic TSHR expressed by the thyroid, is conserved through vertebrate evolution. Both *Tshra* and *Tshrb* are expressed in eel non-thyroid tissues with specific distribution. *Tshra* is expressed in the eye while *Tshrb* is mainly expressed in the pituitary and ovary and also detected in different parts of the brain and adipose tissue as well as some other peripheral tissues (Fig. 7). The similarity of expression profiles between *Tshβ3* and *Tshrb* makes it very tempting to speculate that TSHβ3 might act as a paracrine or autocrine factor of TSHRb.

Non-thyroidal expression was reported for TSHRa in other teleosts, notably in the gonads in catfish [54], [55] and fathead minnow [51], two ostariophysian species that, in contrast to the eel, might possess only this type of receptor. *Tshra* was also found in the gonad of striped bass [56] and European sea bass [57], species which may possess both receptors, according to their phylogenetic position among teleosts. The only available information about the tissue distribution of the b-type receptor is restricted to the truncated transcript of *Tshrb* in the sole, that showed similar expression patterns as classical *Tshra* [20]. It will be interesting to investigate what effect the translocation of *Tshrb* from one genomic region to another has had on control of its expression.

In mammals, in addition to the thyroid follicles, TSHR expression has been described in several tissues notably anterior pituitary, hypothalamus, ovary, testis, skin, immune cells and adipose tissue [58]–[61] but the specific roles in these non-thyroid tissues are not fully known. As compared to the mammalian single TSHR, the tissue distribution of the duplicated eel TSHR highlights a clear sub functionalization, with TSHRa involved in the thyroid control and TSHRb in various non-thyroidal functions.

## Conclusion

**Figure 8:**
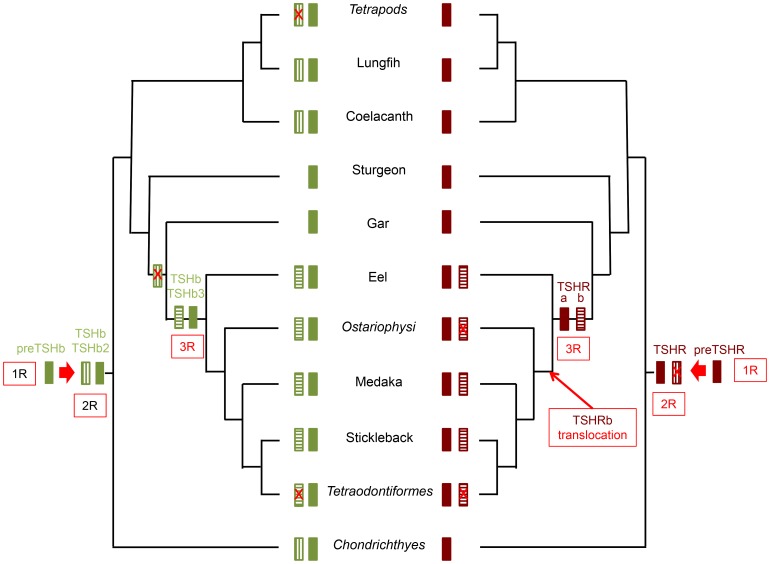
*Tshβ* and*Tshr* gene evolution. *Tshβ* and *Tshβ2* (left panel) were generated by duplication of a *preTshβ* gene through the 2R before the emergence of Gnathostomes. *Tshβ2* was lost (red crossed box) in tetrapods and in ray-finned fish before the emergence of the gar. *Tshβ3* was generated in teleosts by duplication of *Tshβ* through the 3R. Most teleost groups have kept *Tshβ3*. Only one *Tshr* (right panel) is found in vertebrates except in teleosts where a second gene was generated through the 3R. It was translocated to a new genomic environment after the emergence of the eel. Several groups of teleosts have lost this second *Tshr*.

The present study revealed that two TSHs would have arisen from the second global genome duplication (2R) in early vertebrates (Fig. 8), concurrently with the two gonadotropins LH and FSH [14]. The duplicated *Tshβ2* has been conserved in cartilaginous fish and in early lobe-finned fish, but would have been lost both in tetrapods and in early ray-finned fish. This loss of the second *Tshβ* may be related to the redundancy of two TSH, acting via a unique receptor, since the putative duplicated TSH receptor issued from the 2R would have been lost early in the vertebrate evolution (Fig. 8). A second chance for a TSH system doubling occurred in teleosts when a novel global genome duplication (3R) occurred in the lineage. Both duplicated *Tshβ/Tshβ3* and duplicated *Tshra/Tshrb* are found in various extant teleosts, while *Tshβ3* and *Tshrb* may have been lost in some teleost groups. The eel provides a remarkable example of conservation of a duplicated TSH/TSHR system. The endocrine thyrotropic function in the eel would be essentially mediated by the classical TSH and TSHR, which are mainly expressed in the pituitary and thyroid, respectively. The comparison of the distribution pattern of the duplicated *Tshβ3* and the duplicated *Tshrb* shows a striking similarity that could confer a possible autocrine/paracrine role for this couple in several non-thyroidal tissues in the eel. However, the higher evolutionary rate observed in teleosts for the duplicated *Tshβ3* and *Tshrb* suggests that their functions may not be fully stabilized yet. These advances in the evolutionary scenario of TSH and TSHR in vertebrates open new research avenues concerning the functional relationships between the two duplicated TSH and TSHR. Until now little is known about the TSH-TSHR system except in tetrapods. The structural characteristics of duplicated TSHβ in teleost have been well conserved suggesting it might be functional. Further investigations on ligand properties and receptor selectivity and activity are required to evaluate the biological importance of the duplication of the system TSH-TSHR and to infer evolutionary drives that contributed to the maintenance of the duplicated ligand –receptor system.

## Supporting Information

Figure S1 **TSHβ subunit-related sequences alignment.**
(PDF)

Figure S2 **Reconstructed eel genomic regions flanking TSHβ (A) and TSHR (B) genes.**
(PDF)

Figure S3 **TSHR-related sequence alignment.**
(PDF)

Figure S4 **Relative evolution rates between TSHβ and TSHβ3 and between TSHR-a and -b sequences.**
(PDF)

Table S1 **Database references for TSHβ subunit-related sequences.**
(PDF)

Table S2 **Database references for TSHR-related sequences.**
(PDF)

Table S3 **European eel primer sets for quantitative real-time PCR.**
(PDF)

Table S4 **Database references for the genes in TSHβ and TSHR genomic regions.**
(PDF)
