# Supplementary material for: Multiple Thyrotropin β-Subunit and Thyrotropin Receptor-Related Genes Arose during Vertebrate Evolution
Source: PLoS One. 2014 Nov 11;9(11):e111361. doi: 10.1371/journal.pone.0111361 (PMC4227674; doi:10.1371/journal.pone.0111361)
Supplement: Figure S1 — TSHβ subunit-related sequences alignment. (PDF) [file pone.0111361.s001.pdf]

```
TSHβ3 Yellow perch            ------MGSLVVKCMLLCALMHGAVCACMLKNHTIWIERQDCGQCVAINTTICSGYCYTHDTNLRGRLGRSFLIQRSCVPLSLVYRAAHVPGCPQDVSPQLFYPAANRCSCRRCDTRTHHCVRDSR-IPNDRCTVTLGDVKNQTPPSVGTC*--------
TSHβ3 Sablefish               ------MPLLLLKCILLCALMREAVCACMLNNHTLWIERRDCAQCVAINTTICNGYCYTQDTNLRGRFGRSFVIQRSCVPHSLVYRAAHVPGCPQDVNPQLYYPAAHRCSCRRCDTRTHHCVRASR-ISNDRCTVTLGGVKSQTQPSVGT*---------
TSHβ3 Bluefin tuna            ------MCLFVFKCMLLCMLTSGTVCACMLKNHTIWIERHDCAQCVAINTTICSGYCYTQDTNLKGWFGRTFLIQRSCVPLSLVYQAVHFPGCPQNVNPQLYFPVALCCSCRRCDSRTHHCVRSSQ-IPHDRCTMALGSLKNENQPALET*---------
TSHβ3 Flag rockfish           ------MSSSVLKCMLLCALMHGAVCACTLKNHTIWIERQDCAQCVAVNTTICSGYCYTQDTNLRGRFGRSFLIQRSCVPHSLVYRAAHVPGCPQDVNSQLYYPAAQRCSCRRCDTRTHHCVRTRR-ISHDGCTVTLGGVRSQTQPAVGT*---------
TSHβ3 Antarctic toothfish     ---MSRMTSLVLKCMLLCALMHGTVFSCMLKNHTIWIERQDCGQCVAINTTICSGYCHTQDTNLKGRFGRIFLIQRSCVPLSLVYRAVHVPGCPQDVNSQLYYPAAQRCSCRRCNTRSHHCVRTSR-VSKDRCTVTQ???-------------------
TSHβ3 Stickleback             ------MPLLVLKCMLLCALMHRAACACMLSNHTLWIESRDCAQCVAINTTICKGYCYTKDTNLKGRFGRDFMIQRSCVPLSLVYRAVHLPGCPPGVNPQVYYPAAHRCLCKRCDTRTHHCVRTSR-VSTEGCSATLDGVKSQTQPSVATQQYVNTGDAA
TSHβ3 Amazon molly            ------MPLFILRRTLLFALMVGAVYTCTLMNHTIWLEKKNCTQCVAVNTTICSGYCYTRDTNLKGSFGRAFLIQRSCVPISLVYRAVFIPGCPQDVGSQLYYPAARCCSCRRCDTRTHHCVKPRP-YSYDQCSVKLGGVEKQNLKCNAPTCQMAVNQL/
TSHβ3 Southern platyfish      ------MSLFILRWTLLFALMVGAVYTCMLMNHTIWIEKENCTQCVAVNTTICSGYCYTRDTNLKGRFGRAFLIQRSCVPISLVYRAVFIPGCPQDVSSQLYYPAARCCSCRRCDTRTHHCVKPRP-YSYDQCSVELGGMEKQNLKCFGNLTTCS*----
TSHβ3 Burton's mouthbrooder   ------MPLFALKSLLLCVLMVGAVHTCMLKNYTLWIEKQDCTQCVAINTTICSGYCYTQDTNLRGRFGRTFLIQRSCVPLSLVYRAAHIPGCPKDVNPQLYYPAAHCCSCRRCDTRTHRCVRTSR-IPYDQCFTTLDSVKKQNQSAVEISQYSNTSSS/
TSHβ3 Pundamilia              ------MPLFALKSLLLCVLMVGAVYTCMLKNYTLWIEKQDCTQCVAINTTICSGYCYTQDTNLRGRFGRTFLIQRSCVPLSLVYRAAHIPGCPKDVNPQLYYPAAHCCSCRRCDTRTHRCVRTSR-IPYDQCFTTLDSVKKQNQSAVEISQYSNTSSS/
TSHβ3 Zebra mbuna             ------MPLFALKSLLLCVLMVGAVYTCMLKNYTLWIEKQDCTQCVAINTTICSGYCYTQDTNLRGRFGRTFLIQRSCVPLSLVYRAAHIPGCPKDVNPQLYYPAAHCCSCRRCDTRTHRCVRTSR-IPYDQCFTTLDSVKKQNQSAVEISQYSNTSSS/
TSHβ3 Nile tilapia            ------MPLSALKSLLLCALMVGAVYTCMLKNYTLWIEKQDCTQCVAINTTICSGYCYTQDTNLKGRFGRTFLIQRSCVPLSLVYQAAHIPGCPKDVNPQLYYPAAHCCSCRRCDTRTHRCVRTSR-IPYDQCFTTLDSVKKQNQSALEISQYSNTSGS/
TSHβ3 Lyretail cichlid        ------MPLFALKSSLLCVLMVGAVYSCMLKNYTLWIEKQDCTQCVAINTTICSGYCYTQDTNLRGRFGRTFLIQRSCVPLSLVYRAAHIPGCPKDVNPQLYYPAAHCCSCRRCDTRTHRCVRTSR-VPYDQCFTTLDGVRNQNQSAVEISQYSNTSSS/
TSHβ3 Medaka                  ------MSLFMLKSALVLAVMAGTVCACVLKNHTIWVEKQNCTQCIAINTTICSGYCYSRDTNFRGRFGRTFLIQRSCMPLSLVYRVAHIPGCPPDVNAELYYPVAHCCSCRCCDTRTYHCVQPRR-FSYDQCSVKLGSGGHQNENCFGNITNC*-----
TSHβ3 Atlantic cod            MDRVYLSSMSLFVCVLLCVLIADTACGCTLKNFTLMIEKYECEQCVLINTTICSGYCYTQDTNFRGRVGKNFLIQRGCTPGSLVYRTARLVGCPRNVNPVIYYPEFHRCKCRSCDRRTHHCVQKSR-YPLNQCRKTRHKRKGKDSN*-------------
TSHβ3 Atlantic salmon         --------MYVLAWVLLFVWLGGGVCVCMMENYTLLIEKRGCSQCIAVNTTICSGFCHTQD???------------------------------------------------------------------------------------------------
TSHβ3 Mexican tetra           ------MGGSVVLQVLLVSLAAGVLLGCSLKNFTLHVEKQECTHCLTINTTVCSGMCFTQDSNLQGVAGRKFRVQRACVYQSVAYRSAEVPGCPAHIDPLYIYPVAQRCRCSKCNTVTNECVQTPL-QLHDSCRSKQQLQ*-------------------
TSHβ3 Zebrafish               --------MRVLLCSFLLLLGEDALLACSLKNYTLYVEKHECGHCMAINTTVCSGMCFTRDTNVQGFVGKRFLLQQSCMHRSLVYRSARMPGCPVHIDPLFFYPVARRCNCTKCNTSRNECVFRHK-HKHNRCSKQLRTV*-------------------
TSHβ3 European eel            ------MALDSLACVLLCLLLGQALAKCVPQNYTLYVEREGCEHCVAVNTTVCRGFCFSRDTNMKKCGLKGFPVQRACMYQSLVYHAVSLPGCLPDVDPLFSFPVALRCHCSRCNTSNTECLHRGK-RLPSPCDSTLCYAKAPPKAATAASLTETFQEN/
TSHβ European eel             ------MRVVLLASAVLCLLAGQVLSICSPVDYTLYVEKPECDFCVAINTTICMGFCYSLDPNVVGPAVKRLVVQRGCTYQAVEYRTAELPGCPPHVDPRFSYPVALHCTCRACDPARDECTHRAS-ADGDRCSKPLLLHMHAYPGQSNYIQTL*-----
TSHβ Zebrafish                -------MSLLYVIGMLGLLMKVAVPMCAPTDYTIYIERQECNYCVAVNTTICMGFCFSRDSNIKELVGPRFIVQRGCTYQEVEYRTAVLPGCPSHADPHFTYPVALSCHCSTCKTHSDECALRTR-SAGMRCSKPVH-HLYPE--ENNYAQAYWDQYE*
TSHβ Mexican tetra            ------MSATVLVAGILGLLLKTAMPMCTPTEYTIYIDKQECDYCVAVNTTICMGFCFSRDSNMKELVGPRFLIQRSCTYQKVEHRTAVLPGCPPHVDPHFTYPVALSCHCSMCNTHSDDCSHKGN-SALAKCSKPVR-PLYPDPAQNDLLQPDWLQLF*
TSHβ Atlantic salmon          ------MELSVAMCGLLCLLFSQAVPMCVPTDYTLYEERRECDFCVAINTTICMGFCYSRDSNMKELAGPRFLIQRGCTYDQVEYRTVILPGCPLHANPLFTYPVALSCHCGTCNTDSDECAHKASSGDGARCSKPLR-HIYPYPGLNNYIPPN*-----
TSHβ Atlantic cod             ------MDYFVFVGSVLLLMFSPAAPMCVPTDYTLYVEKPECNFCVAINTTICMGFCYSRDSNIGDLVGLRFLLQRGCTYDKVEYRAALLPGCPIDSDPVFSYPVALSCRCGTCRTDSDECVHRAPGVGGARCTKPVR-RIYPYPGQSTYMTPF*-----
TSHβ Medaka                   ------MNTVLFPFWMLFLLLSPVVPMCLPTDFTLYVEKPECDYCVAVNTTICTGFCYSRDSNMRDIFGPRFLIQRGCTYDKVEYRTAILPGCPINANPVFTYPVALSCHCGACRTDSDECAHRTS-VDGARCTKPVR-RIYPYPGQSNYVIPF*-----
TSHβ Lyretail cichlid         ------MEATVFNCWLFFLLFSPAVPMCLPTDFTLYVEKPECEFCVAINTTICMGFCYSRDSNMRDILGPRFLVQRGCTYDKVEYHTAILPGCPIEANPVFTYPVALSCHCSACRTDTDECAHRAS-MDGTKCTKPVR-RIYPYPGHSNYVIPF*-----
TSHβ Nile tilapia             ------MEATVFNCWLFFLMFSPAVPMCLPTDFTLYVEKPECEFCVAINTTICMGFCYSRDSNMRDILGPRFLVQRGCTYDKVEYHTAILPGCPIEANPVFTYPVALSCHCSACRTDTDECAHRAS-MDGTKCTKPVR-RIYPYPGHSNYVIPF*-----
TSHβ Zebra mbuma              ------MEVTVFNCWLFFLMFSPAVPMCLPTDFTLYVEKPECEFCVAINTTICMGFCYSRDSNMRDILGPRFLVQRGCTYDKVEYHTAILPGCPIEANPVFTYPVALSCHCSACRTDTDECAHRAS-MDGTKCTKPVR-RIYPYPGHSNYVIPF*-----
TSHβ Pundamilia               ------MEVTVFNCWLFFLMFSPAVPMCLPTDFTLYVEKPECEFCVAINTTICMGFCYSRDSNMRDILGPRFLVQRGCTYDKVEYHTAILPGCPIEANPVFTYPVALSCHCSACRTDTDECAHRAS-MDGTKCTKPVR-RIYPYPGHSNYVIPF*-----
TSHβ Burton's mouthbrooder    ------MEVTVFNCWLFFLMFSPAVPMCLPTDFTLYVEKPECEFCVAINTTICMGFCYSRDSNMRDILGPRFLVQRGCTYDKVEYHTAILPGCPIEANPVFTYPVALSCHCSACRTDTDECAHRAS-MDGTKCTKPVR-RIYPYPGHSNYVIPF*-----
TSHβ Southern platyfish       ------METSAFSCWVLFLLIYPVVPMCLPTEFTLFVEKPECDYCVAINTTICMGVCFTRDSNMRDIFRSRFVVQRSCTYDKVEYRSAILPGCPLESNPVFTYPVALSCHCSACRTDSDECTHRAS-TGGGRCTKPVR-LVHPYPGQSTYMILF*-----
TSHβ Amazon molly             ------METSAFSCWVLFLLIYPVVPMCLPTESTLFVEKPECDYCVAVNTTICMGVCFTRDSNIGDIFHSRFVFQRSCTYDKVEYRTVILPGCAIDSNPAYTYPVAISCHCGACRTDRDECTLRLN-SYDANCAKPVR-RVYPYPGQSNYMIPF*-----
TSHβ Stickleback              ------METAVFPCWLLFLLLSPAVPTCFPTDFTMYVERPECDYCVAINTTICMGFCYSRDSNVRAIVGPRFLIQTGCNYDKVEYRTVILPGCAIGSNPAYTYPVAISCHCGACRTERDECTHRVN-VYDANCAKPVR-RVYPYPGQSNYMIPF*-----
TSHβ Flag rockfish            ------METAVFTCWLLFLLFSPAVPMCLPTDFTLYVERPECDYCVAINTTICMGFCYSRDSNMRDIIGPRFLIQRGCTYDKVEYRTAILPGCPIDTNAVFTYPVALSCHCGACRTDSDECAHRAS-VDGARCTKPVR-RIYPYPGQSNYMIPF*-----
TSHβ Bluefin tuna             -----MTDTAMFTCWLLFLLFSPAVPMCLPTDFTLYVEKPECDFCVAINTTICMGFCYSRDSNMRDILGPRFLIQKGCTYNQVEYRTAILPGCPSEGSSLFSYPVALSCHCGACNTAVDECAHRAS-SNRPTCTKPVR-HIY----QSNFLLPF*-----
TSHβ Sablefish                ------METAVFTCWLLFLLFSPAVPMCFPTDFTLYVERPECDYCVAINTTICMGFCYSRDSNVRAIVGPRFLIQTGCTYDKVEYRAAILPGCPVDSDPVFSYPVALSCHCGACRTESDECAHRAS-MGGPRCTKPVR-RIYPYPGQTSYMTPL*-----
```

Overall conserved amino acids are in red. Conserved positions that differ between the two sister gene sequences are highlighted. Potential N-glycosylation sites are in bold type.
