# Supplementary material for: Multiple Thyrotropin β-Subunit and Thyrotropin Receptor-Related Genes Arose during Vertebrate Evolution
Source: PLoS One. 2014 Nov 11;9(11):e111361. doi: 10.1371/journal.pone.0111361 (PMC4227674; doi:10.1371/journal.pone.0111361)
Supplement: Figure S2 — Reconstructed eel genomic regions flanking TSHβ (A) and TSHR (B) genes. (PDF) [file pone.0111361.s002.pdf]

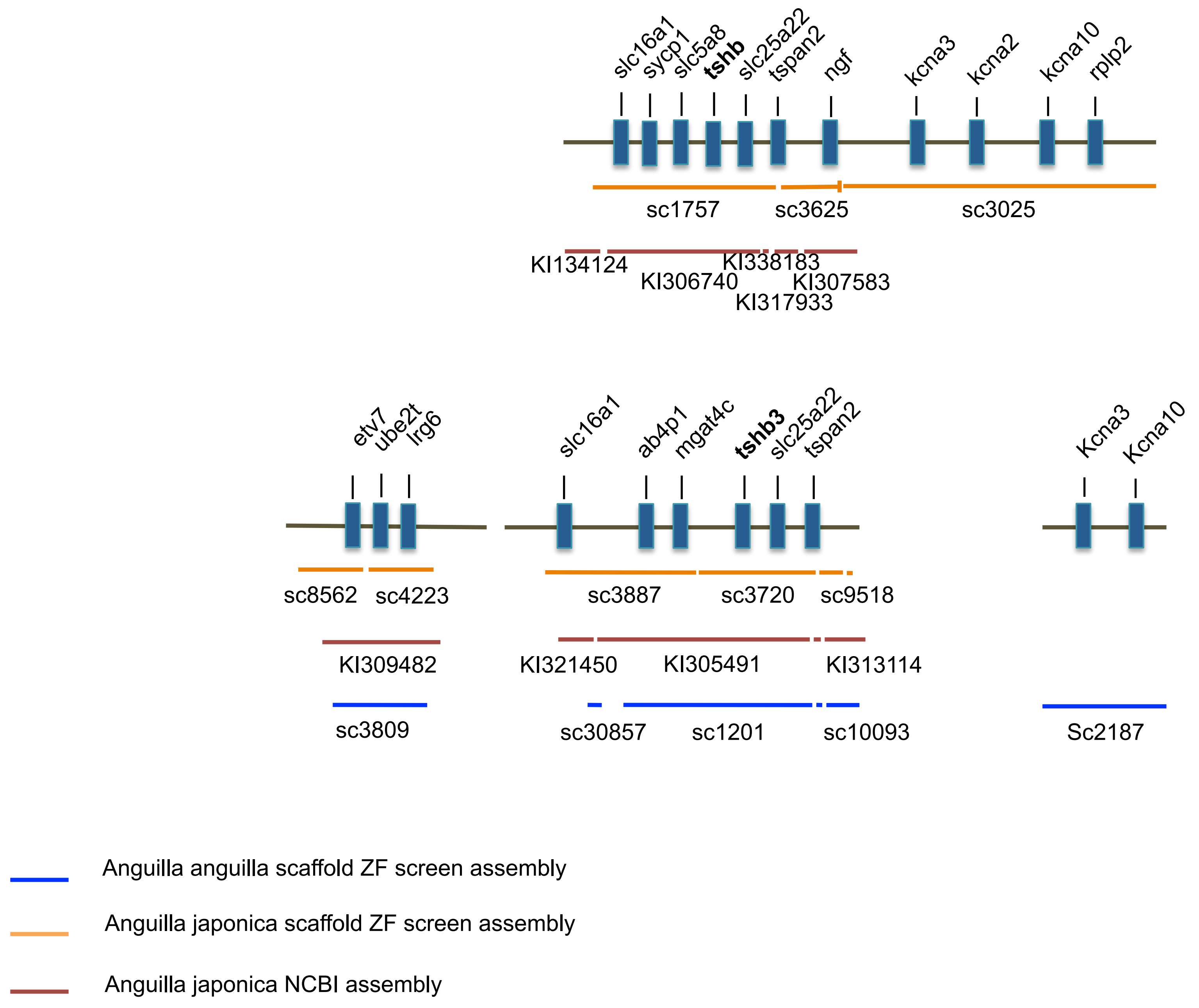
slc16a1
sycp1
slc5a8
tshb
slc25a22
tspan2
ngf
kcna3
kcna2
kcna10
rplp2
sc1757
sc3625
sc3025
KI134124
KI338183
KI306740
KI307583
KI317933
etv7
ube2t
lrg6
slc16a1
ab4p1
mgat4c
tshb3
slc25a22
tspan2
Kcna3
Kcna10
sc8562
sc4223
sc3887
sc3720
sc9518
KI309482
KI321450
KI305491
KI313114
sc3809
sc30857
sc1201
sc10093
Sc2187
Anguilla anguilla scaffold ZF screen assembly
Anguilla japonica scaffold ZF screen assembly
Anguilla japonica NCBI assembly

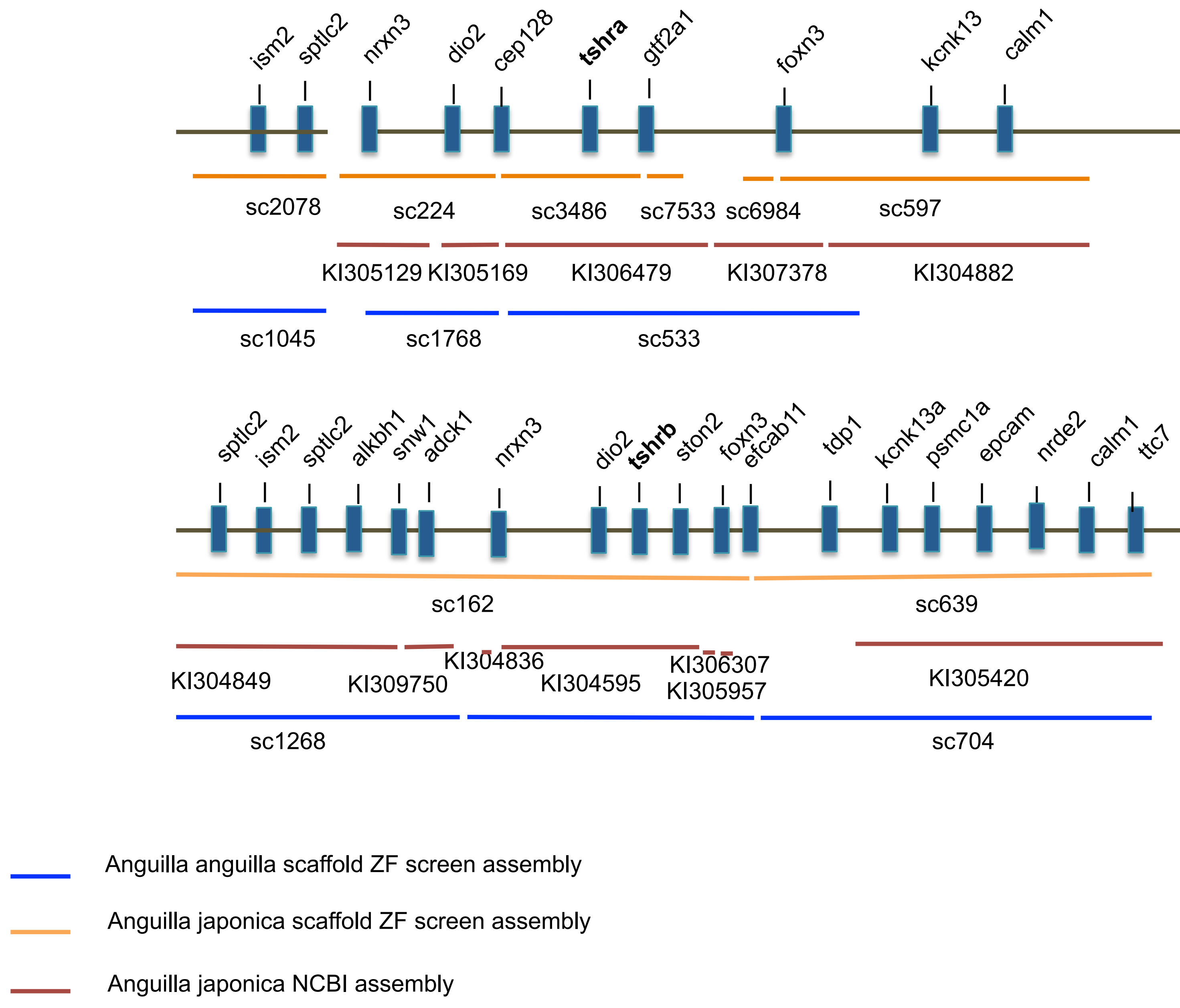

ism2
sptlc2
nrxn3
dio2
cep128
tshra
gtf2a1
foxn3
kcnk13
calm1
sc2078
sc224
sc3486
sc7533
sc6984
sc597
KI305129
KI305169
KI306479
KI307378
KI304882
sc1045
sc1768
sc533
sptlc2
ism2
sptlc2
alkbh1
snw1
adck1
nrxn3
dio2
tshrb
ston2
foxn3
efcab11
tdp1
kcnk13a
psmc1a
epcam
nrde2
calm1
ttc7
sc162
sc639
KI304849
KI309750
KI304836
KI304595
KI306307
KI305957
KI305420
sc1268
sc704
Anguilla anguilla scaffold ZF screen assembly
Anguilla japonica scaffold ZF screen assembly
Anguilla japonica NCBI assembly
