# Supplementary material for: Multiple Thyrotropin β-Subunit and Thyrotropin Receptor-Related Genes Arose during Vertebrate Evolution
Source: PLoS One. 2014 Nov 11;9(11):e111361. doi: 10.1371/journal.pone.0111361 (PMC4227674; doi:10.1371/journal.pone.0111361)
Supplement: Figure S3 — TSHR-related sequence alignment. (PDF) [file pone.0111361.s003.pdf]

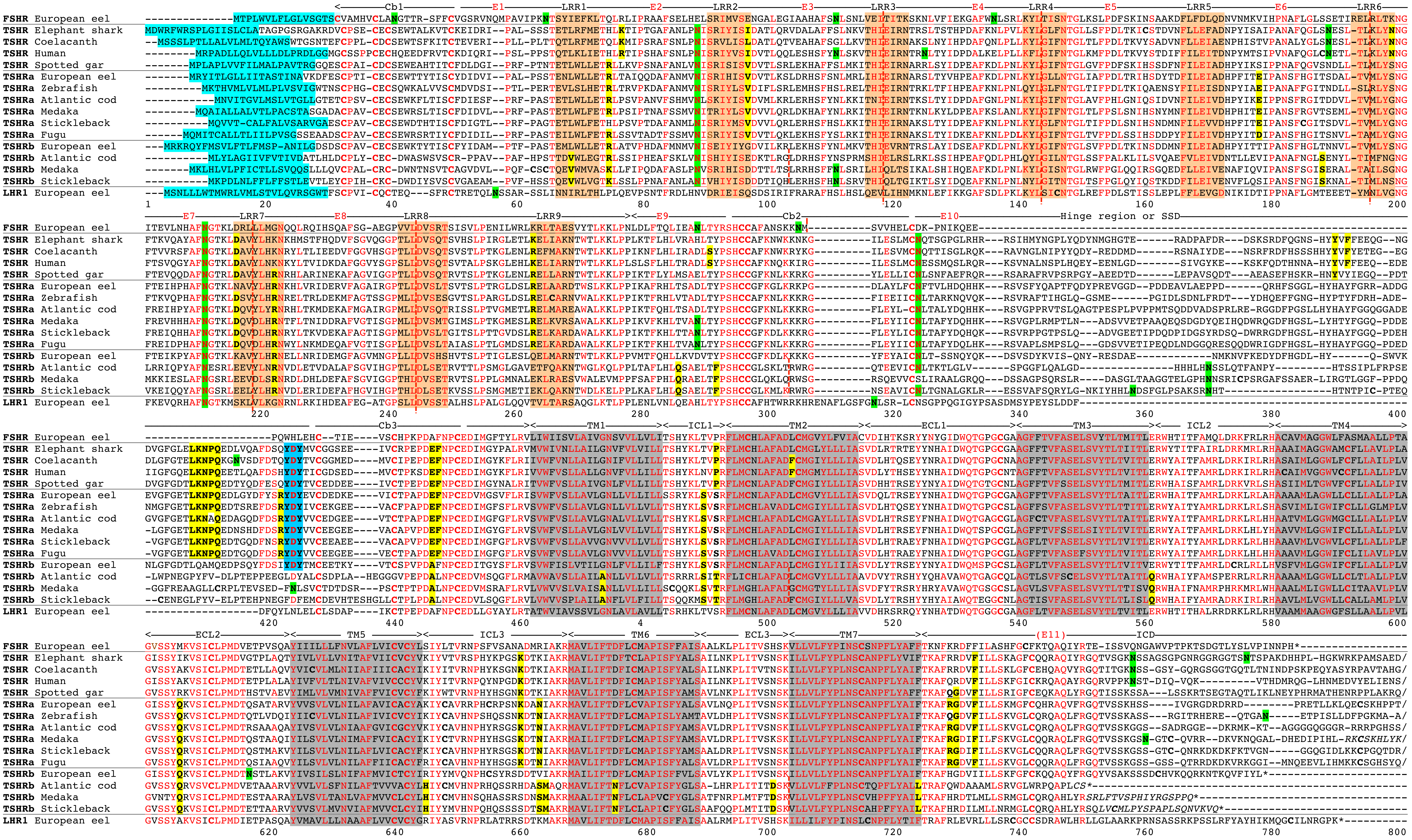

Fig S3: Amino acid alignment of representative sequences of the two TSHR types. Eel FSHR and LHR1 are given for comparison. Highly conserved amino acids are in red. Potential N-linked glycosylation sites are in green background. The potential sulfation site is in blue background. LRR motifs are indicated with orange background. Predicted alpha helical transmembrane domains are shaded in grey. Type-specific positions are in bold and highlighted in yellow. Cysteine residues are in bold. Predicted signal peptide is highlighted in blue. Exons (indicated on the top line in red) are delineated by vertical dotted red lines. Uncertain carboxy-terminal sequences (depending on the presence or not of an additional exon) are in italics. Sequences might be truncated at the carboxy-terminal end for convenience (/).
