# Supplementary material for: Multiple Thyrotropin β-Subunit and Thyrotropin Receptor-Related Genes Arose during Vertebrate Evolution
Source: PLoS One. 2014 Nov 11;9(11):e111361. doi: 10.1371/journal.pone.0111361 (PMC4227674; doi:10.1371/journal.pone.0111361)

	TSHb	TSHb3		TSHRa	TSHRb
eel	37	43	eel	48	61
zebrafish	54	56	Atl. salmon	32	113
Mex. tetra	49	79	Atl. cod	57	175
Atl. cod	49	82	medaka	65	203
rockfish	41	77	platy	61	184
lyretail	51	88	sole	51	184
tilapia	51	87	tilapia	50	187
molly	62	96	stickleback	51	171
medaka	56	96	Burton	50	188
tuna	44	80	molly	66	186
stickleback	52	87	damselfish	46	168
sabrefish	45	79			

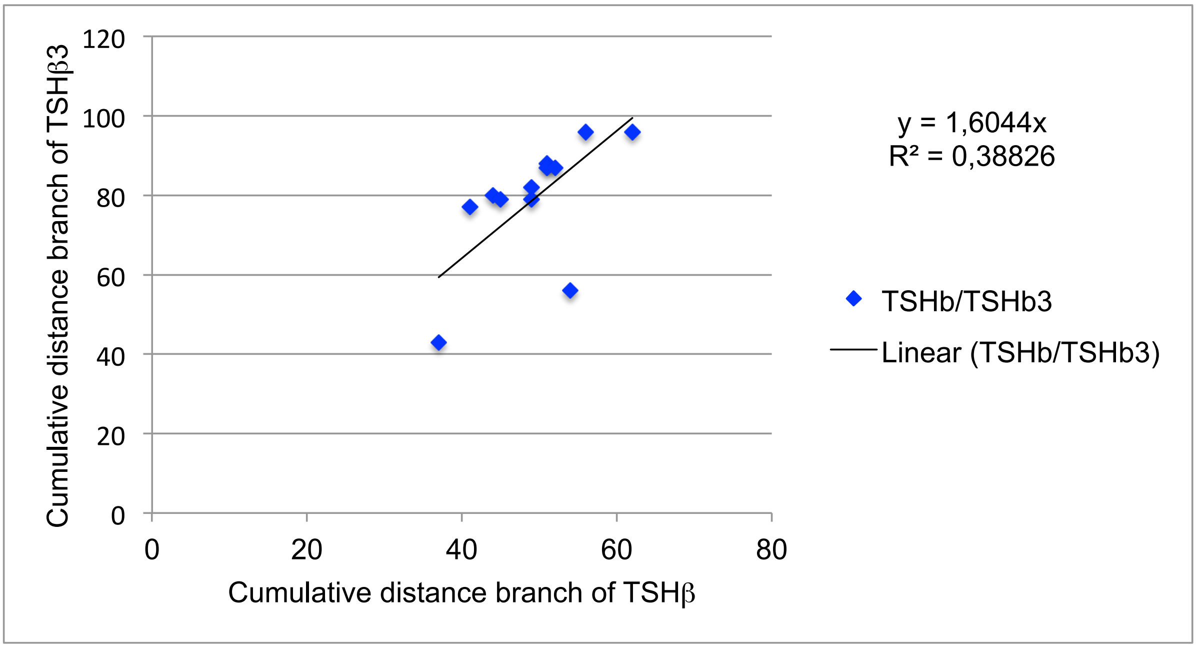

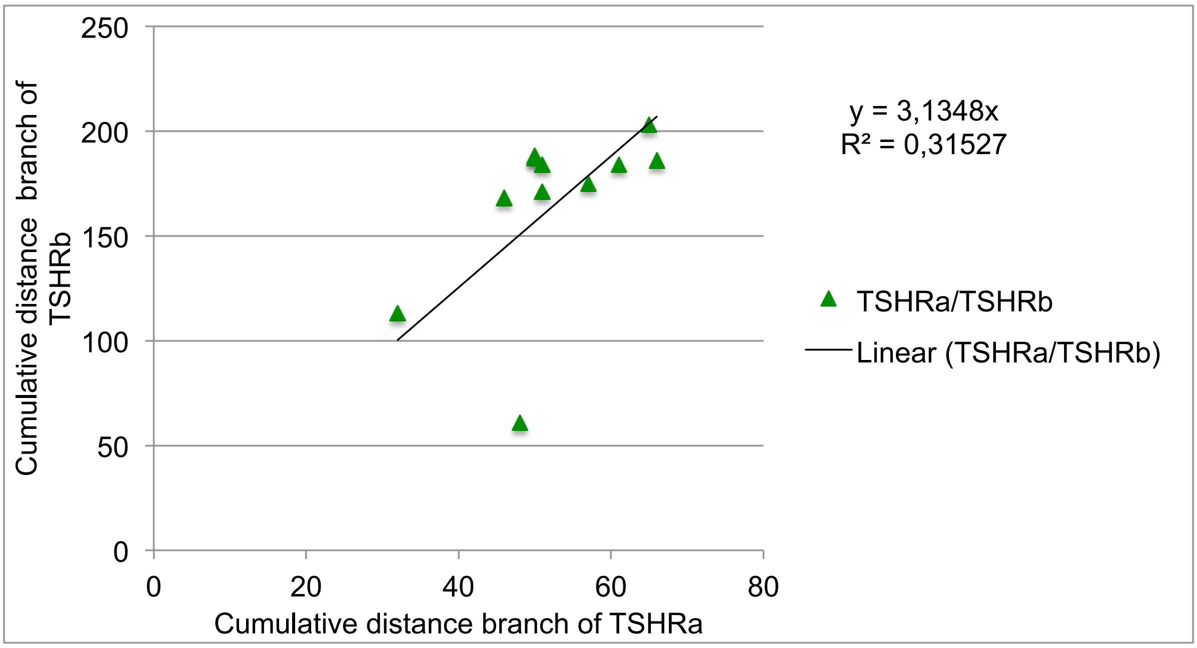

Supplement: Figure S4 — Relative evolution rates between TSHβ and TSHβ3 and between TSHR-a and -b sequences. (PDF) [file pone.0111361.s004.pdf]
