# Supplementary material for: Multiple Thyrotropin β-Subunit and Thyrotropin Receptor-Related Genes Arose during Vertebrate Evolution
Source: PLoS One. 2014 Nov 11;9(11):e111361. doi: 10.1371/journal.pone.0111361 (PMC4227674; doi:10.1371/journal.pone.0111361)
Supplement: Table S1 — Database references for TSHβ subunit-related sequences. (PDF) [file pone.0111361.s005.pdf]

species common name	Binomial nomenclature	Gene	source	exon 1	database reference	coordinates	exon 2	coordinates	Gene	source	exon 1	database reference	coordinates	exon 2	reference if different	coordinates
Flag rockfish	*Sebastes rubriventus*	TSHb	genomic	1-162	AUPQ01064924.1	minus 5189..5028	163-441	plus 3096..2816	TSHb3	genomic	1-162	AUPQ01006956.1	plus 2771..2932	163-432	AUPQ01006959.1	plus 161..430
Bluefin tuna	*Thunnus orientalis*	TSHb	genomic	1-165	BADN01002036.1	minus 12234..12070	166-444	minus 11290..11012	TSHb3	genomic	1-162	BADN01031353.1	minus 15072..14911	163-432		minus 14535..14266
Sablefish	*Anoplopoma fimbria*	TSHb	genomic	1-162	AWGY01180279.1	minus 845..684	163-441	minus 390..112	TSHb3	genomic	1-162	AWGY01127952.1	plus 471..632	163-432		plus 1235..1504
Stickleback	*Gasterosteus aculeatus*	TSHb	genomic	1-162	AANH01007474.1	minus 57268..57107	163-444	minus 56925..56644	TSHb3	genomic	1-162	AANH01002636.1	plus 80149..80310	163-462		plus 80748..81047
Amazon molly	*Poecilia formosa*	TSHb	genomic	1-162	AYCK01002369.1	plus 78070..78231	163-441	plus 78726..7904	TSHb3	genomic	1-162	AYCK01012762.1	minus 104341..104180	163-447		minus 10359..103313
Southern platifish	*Xiphophorus maculatus*	TSHb	genomic	1-162	AGAJ01036774.1	plus 27010..27171	163-441	plus 27572..27850	TSHb3	genomic	1-162	AGAJ01024990.1	minus 11856..11696	163-447		minus 11184..10901
Burton's mouthbrooder	*Haplochromis burtoni*	TSHb	genomic	1-162	AFNZ01035994.1	minus 4746..4585	163-441	minus 4004..3726	TSHb3	genomic	1-162	AFNZ01002187.1	plus 7732..7893	163-474		plus 8140..8451
Pundamilia	*Pundmilia nyererei*	TSHb	genomic	1-162	AFNX01016941.1	minus 18633..18472	163-441	minus 17891..17613	TSHb3	genomic	1-162	AFNX01016941.1	plus 10741..10902	163-474		plus 111448..111459
Zebra mbuna	*Maylandia zebra*	TSHb	genomic	1-162	AGTA02011887.1	minus 9243..9082	163-441	minus 8501..8223	TSHb3	genomic	1-162	AGTA02027934.1	minus 34202..34043	163-474		minus 33795..33485
Nile tilapia	*Oreochromis niloticus*	TSHb	EST		GR610467				TSHb3	genomic	1-162	AERX01019473.1	plus 43620..43459	163-474		plus 43200..42889
Lyretail cichlid	*Neolamprologus brichardi*	TSHb	genomic	1-162	AFNY01008351.1	plus 29482..29643	163-441	plus 30247..30225	TSHb3	genomic	1-162	AFNY01015949.1	minus 9583..9422	163-474		minus 9195..8884
Medaka	*Oryzias latipes*	TSHb	genomic	1-162	BAAF04092333.1	minus 6778..6617	163-441	minus 5623..5345	TSHb3	genomic	1-162	BAAF04002594.1	minus 9769..9608	163-444		minus 9426..9145
Atlantic cod	*Gadus morhua*	TSHb	genomic	1-162	CAEA01180393.1	minus 734..573	163-429	minus 295..29	TSHb3	genomic	1-162	CAEA01110666.1	minus 2083..1904	163-432		minus 1768..1511
Atlantic salmon	*Salmo salar*	TSHb	mRNA		AF060566.1				TSHb3	genomic	1-157	AGKD01234435.1	plus 1248..1404	not found		
Mexican tetra	*Astyanax mexicanus*	TSHb	genomic	1-162	APWO01099122.1	minus 33860..33699	163-456	minus 32724..32431	TSHb3	genomic	1-162	APWO01049929.1	plus 702..863	163-402		plus 3344..3583
Zebrafish	*Danio rerio*	TSHb	mRNA		AY135147.1				TSHb3	genomic	1-156	CAbZ01052937.1	plus 1294..1449	157-396		plus 2670..3209
European eel	*Anguilla anguilla*	TSHb	mRNA		X73493.1				TSHb3	genomic	1-162	scaffold1201	minus 46264..46103	163-498		minus 43490..43155
Japanese eel	*Anguilla japonica*	TSHb	mRNA		AY158008.1				TSHb3	genomic	1-162	AVPY01071335.1	minus 859..698	163-498	AVPY01071334.1	plus 2290..1955
Elephant shark	*Callorhinchus millii*	TSHb	mRNA		HQ174785.1				TSHb2	mRNA		HQ174784.1				
Coelacanth	*Latimeria chalumnae*	TSHb	mRNA		XM_005988466.1				TSHb2	mRNA		XM_006002575.1				
Little skate	*Leucoraja erinacea*								TSHb2	genomic	1-162	AESE012561233.1	minus 411..572	163-480	AESE011500728.1	plus 164-481
Australian lungfish	*Neoceratodus forsteri*								TSHb2	mRNA		AJ578039.1				
Japanese toad	*Bufo Japonicus*	TSHb	mRNA		AB085671.1											
Bullfrog	*Rana catesbeiana*	TSHb	mRNA		AB443444.1											
African clawed frog	*Xenopus tropicalis*	TSHb	mRNA		XM_002942789.2											
Opossum	*Monodelphis domestica*	TSHb	mRNA		AY048589.1											
Cattle	*Bos taurus*	TSHb	mRNA		K01939.1											
Human	*Homo sapiens*	TSHb	mRNA		M21024.1											
Mouse	*Mus musculus*	TSHb	mRNA		J00644.1											
Platipus	*Ornithorhynchus_anatinus*	TSHb	mRNA		XM_001506079.2											
Chicken	*Gallus gallus*	TSHb	mRNA		AF033495.1											
Crested ibis	*Nipponia nippon*	TSHb	mRNA		AB089501.1											
Alligator	*Alligator mississippiensis*	TSHb	mRNA		XM_006260749.1											
Chinese soft-shelled turtle	*Pelodiscus sinensis*	TSHb	mRNA		AY618874.1											
Painted turtle	*Chrysemys picta*	TSHb	mRNA		XM_005294934.1											
Green anole	*Anolis carolinensis*	TSHb	mRNA		XM_003220505.2											
Burmese python	*Python molurus bivittatus*	TSHb	genomic	1-168	AEQU02075326.1	plus 706..873	169-399	2832..3064								
King cobra	*Ophiophagus hannah*	TSHb	genomic	1-168	AZIM01004308.1	plus 71164..71331	169-399	73511..73741								
Spotted gar	*Lepisosteus oculatus*	TSHb	mRNA		ENSLOCT00000013043											
Siberian sturgeon	*Acipenser baeri*	TSHb	mRNA		AJ251659.1											
Sea lamprey	*Petromyzon marinus*	GTHb	mRNA		AY730276.1											
