# Supplementary material for: Multiple Thyrotropin β-Subunit and Thyrotropin Receptor-Related Genes Arose during Vertebrate Evolution
Source: PLoS One. 2014 Nov 11;9(11):e111361. doi: 10.1371/journal.pone.0111361 (PMC4227674; doi:10.1371/journal.pone.0111361)
Supplement: Table S2 — Database references for TSHR-related sequences. (PDF) [file pone.0111361.s006.pdf]

<table>
<tr><th>Species name</th><th>Binomial nomenclature</th><th>gene</th><th>source</th><th></th><th>gene / mRNA reference</th><th>Sequence prediction</th><th>Protein reference</th></tr>
<tr><td>Elephant shark</td><td>Callorhinchus milii</td><td>tshr</td><td>genomic</td><td>full-length (10 exons)</td><td>gb|KI636007|</td><td>complement(join(275302..276713,277509..277697,281970..282047,283255..283323,284039..284116,284564..284638,285377..285451,287355..287429,288151..288222,294043..294233))</td><td></td></tr>
<tr><td>Little skate</td><td>Leucoraja erinacea</td><td>tshr</td><td>genomic</td><td>partial (9 exons)</td><td>(1) AESE011610528 (ex 1) / (2) AESE010675100 (ex 3) / (3) AESE011154409 (ex 4) / (4) AESE01113030 (ex 5) / (5) AESE0111796922 (ex 6) / (6) AESE011824310 (ex 7) / (7) AESE010456053 (ex 8) / (8) AESE011861053 (ex 9)/ (9) AESE010365400 (ex 10 start)/ (10) AESE010259803 (ex 10 end)</td><td>(1) 661..833 / (2) 147..218 /(3) 116..190 / (4) 149..223 /(5) complement(7..81) / (6) 109..186 / ex8 complement(144..221) /(8) 43..231 / (9) (1..621) /(10) complement(1..353)</td><td></td></tr>
<tr><td>Coelacanth</td><td>Latimeria chalumnae</td><td>tshr</td><td>genomic</td><td>full-length</td><td>gb NW_005819145</td><td>complement(join(1600975..1602403,1608983..1609171,1622583..1622660,1626096..1626164,1627361..1627438,1634493..1634567,1635332..1635406,1657715..1657789,1671628..1671699,1693546..1693730))</td><td>XP_005992780</td></tr>
<tr><td>Western clawed frog</td><td>Xenopus tropicalis</td><td>tshr</td><td>genomic</td><td>full-length</td><td>gb AAMC02030077</td><td></td><td>XP_002938419</td></tr>
<tr><td>Green anole</td><td>Anolis carolinensis</td><td>tshr</td><td>genomic</td><td>full-length</td><td></td><td></td><td>XP_003214472</td></tr>
<tr><td>Burmese python</td><td>Python bivittatus</td><td>tshr</td><td>genomic</td><td>full-length</td><td></td><td></td><td>XP_007432806</td></tr>
<tr><td>Painted turtle</td><td>Chrysemys picta</td><td>tshr</td><td>genomic</td><td>full-length</td><td></td><td></td><td>XP_005311219</td></tr>
<tr><td>Chinese soft-shelled turtle</td><td>Pelodiscus sinensis</td><td>tshr</td><td>genomic</td><td>full-length</td><td></td><td></td><td>XP_006116039</td></tr>
<tr><td>Zebra finch</td><td>Taeniopygia guttata</td><td>tshr</td><td>genomic</td><td>full-length</td><td></td><td></td><td>XP_002200581</td></tr>
<tr><td>Chicken</td><td>Gallus gallus</td><td>tshr</td><td>genomic / mRNA</td><td>full-length</td><td>NM_001193588</td><td></td><td>NP_001180517</td></tr>
<tr><td>Alligator</td><td>Alligator mississippiensis</td><td>tshr</td><td>genomic</td><td>full-length</td><td></td><td></td><td>XP_006260928</td></tr>
<tr><td>Platypus</td><td>Ornithorhynchus anatinus</td><td>tshr</td><td>genomic</td><td>partial (9 exons)</td><td></td><td></td><td>XP_001505603</td></tr>
<tr><td>Opossum</td><td>Monodelphis domestica</td><td>tshr</td><td>genomic</td><td>full-length</td><td></td><td></td><td>XP_001373592</td></tr>
<tr><td>Human</td><td>Homo sapiens</td><td>tshr</td><td>genomic / mRNA</td><td>full-length (10 exons)</td><td>NG_009206</td><td>join(5157..5326,111624..111695,117730..117804,137430..137504,140545..140619,142007..142084,146115..146183,157851..157928,189155..189343,192416..193829)</td><td>P16473</td></tr>
<tr><td>Rat</td><td>Rattus norvegicus</td><td>tshr</td><td>genomic / cDNA</td><td>full-length</td><td></td><td></td><td>NP_037020</td></tr>
<tr><td>Mouse</td><td>Mus musculus</td><td>tshr</td><td>genomic / cDNA</td><td>full-length</td><td></td><td></td><td>NP_035778</td></tr>
<tr><td>Cattle</td><td>Bos taurus</td><td>tshr</td><td>genomic / cDNA</td><td>full-length</td><td></td><td></td><td>NP_776631</td></tr>
<tr><td>Pig</td><td>Sus scrofa</td><td>tshr</td><td>genomic / cDNA</td><td>full-length</td><td></td><td></td><td>NP_999462</td></tr>
<tr><td>Spotted gar</td><td>Lepisosteus oculatus</td><td>tshr</td><td>genomic</td><td>full-length (10 exons)</td><td>NW_006269932 (LOC102687834)</td><td>join(2519719..2519888,2528264..2528335,2530476..2530550,2533138..2533212,2533783..2533857,2534164..2534241,2534701..2534769,2536787..2536864,2539247..2539435,2539872..2541309)</td><td>XP_006632332</td></tr>
<tr><td>European eel</td><td>Anguilla anguilla</td><td>tshra</td><td>genomic / cDNA partially cloned</td><td>full-length (10 exons)</td><td>scaffold 533</td><td>join(18337..18506,29418..29489,31704..31778,33561..33635,34120..34194,35236..35313,36027..36095,39123..39200,40447..40635,41752..41971,42033..42563,42588..43229)</td><td></td></tr>
<tr><td>Japanese eel</td><td>Anguilla japonica</td><td>tshra</td><td>genomic</td><td>full-length (10 exons)</td><td>gb|KI306479.1|</td><td>join(49771..49940,60345..60416,62429..62503,64331..64405,64827..64901,65948..66025,66741..66809,69868..69945,71211..71399,72515..73907)</td><td></td></tr>
<tr><td>Mexican tetra</td><td>Astyanax mexicanus</td><td>tshra</td><td>genomic</td><td>full-length (10 exons)</td><td></td><td></td><td>XP_007255743</td></tr>
<tr><td>Zebrafish</td><td>Danio rerio</td><td>tshra</td><td>genomic</td><td>full-length (10 exons)</td><td>NC_007131</td><td>join(1..362,12535..12606,16943..17017,20231</td><td>NP_001139235</td></tr>
<tr><td>Atlantic salmon</td><td>Salmo salar</td><td>tshraa</td><td>genomic</td><td>full-length (13 exons/12 exons)</td><td>gb|AGKD03022066.1|</td><td>complement(join(62860..63488,63680..64261,64385..64594,64667..64727,69381..69569,71725..71802,73406..73474,73969..74046,78610..78684,79073..79147,81344..81418,85750..85821,95570..95763))/alternative complement(join(62860..63488,63680..64261,64385..64727,69381..69569,71725..71802,73406..73474,73969..74046,78610..78684,79073..79147,81344..81418,85750..85821,95570..95763))</td><td></td></tr>
</table>

Atlantic salmon	*Salmo salar*	tshrab	genomic	full-length (12 exons)	(1) gb|AGKD03067207.1 | (ex 1-8) // (2) gb|AGKD03065966.1| (ex 9-12)	(a) (1) complement(join(2110..2187,4723..4791,5278..5355,5628..5702,5917..5991,8869..8943,11387..11458,20162..20355))/ (2) join(10019..10206,13036..13285,14582..15163,15288..15908)	
Amago salmon	*Oncorhynchus rhodurus*	tshraa	mRNA	full-length			BAB07800
Amago salmon	*Oncorhynchus rhodurus*	tshrab	mRNA	full-length			BAB07801
Atlantic cod	*Gadus morhua*	tshra		full-length (10 exons)	ENS gadMor1:GeneScaffold_326:79400:103[illegible]	complement(join(134..1754,3697..3885,7698..7775,9712..9780,11107..11184,11451..11525,11762..11836,16263..16337,17140..17211,24137..24306))	ENSGMOP00000017055
Medaka	*Oryzias latipes*	tshra	genomic	full-length (10 exons)	ASM31367v1	join(3491929..3492095,3499343..3499414,3499765..3499839,3501625..3501699,3501946..3502020,3502287..3502364,3503297..3503365,3504698..3504775,3507573..3507761,3507948..3509414)	
Amazon molly	*Poecilia formosa*	tshra	genomic	full-length (10 exons)	AYCK01011186	complement(join(40352..41789,43260..43448,46538..46615,51446..51514,51990..52067,52734..52808,53417..53491,56874..56948,58070..58141,74319..74566))	
Southern platyfish	*Xiphophorus maculatus*	tshra	genomic	full-length (10 exons)			XP_005809988
Senegalese sole	*Solea senegalensis*	tshra	mRNA	full-length			emb| CBK38913|
Stickleback	*Gasterosteus aculeatus*	tshra	genomic (10 exons)	full-length (10 exons)	ENS BROADS1:groupXVIII:2160000:2176400:1 (ENSGACG00000005233)	complement(join(663..2102,2514..2702,5278..5355,6624..6692,7114..7191,8464..8538,9004..9078,11297..11371,12189..12260,16039..16196))	ENSGACP00000006964
Bicolor damselfish	*Stegastes partitus*	tshra	genomic				XP_008282301
Nile tilapia	*Oreochromis niloticus*	tshra	genomic / EST	full-length (10 exons)	NC_022213 /EMBL BAB39132	join(24385861..24386030,24393169..24393240,24394048..24394122,24398384..24398458,24399801..24399875,24400505..24400582,24401159..24401227,24404404..24404481,24409338..24409526,24409892..24411347)	
Burton's mouthbrooder	*Haplochromis burtoni*	tshra	genomic				XP_005917137
Torafugu	*Takifugu rubripes*	tshra	genomic	full-length (10 exons)			XP_003971550
Spotted green pufferfish	*Tetraodon nigroviridis*	tshra	genomic	full-length (10 exons)	emb|CAAE01014590.1|	join(741056..741228,744453..744524,745355..745429,747291..747365,747626..747700,748089..748166,748415..748483,749495..749572,750871..751059,751237..752692)	
European eel	*Anguilla anguilla*	tshrb	genomic / mRNA partially cloned	full-length (10 exons)	scaffold 65	complement(join(336011..337355,339941..340129,343191..343268,345111..345179,350643..350720,353938..354012,354182..354256,359565..359639,362941..363012,364153..364331))	
Japanese eel	*Anguilla japonica*	tshrb	genomic	full-length (10 exons)	gb|KI304595.1|	join(50955..51133,52326..52397,55544..55618,59614..59688,59945..60019,64503..64580,69843..69911,71642..71719,74769..74957,77597..78941)	
Atlantic salmon	*Salmo salar*	tshrb	genomic	partial (11 exons)	gb|AGKD03002913.1| (ex1-5)/ gb|AGKD03002913.1| (ex 6-9) /gb|AGKD03092249.1| (ex 10-11)	complement(join(1991..2065,2699..2773,12486..12560,12793..12864,13531..13703)) / complement(join(77045..77233,78570..78647,79323..79391,79745..79822))/ join(6984..7923,8404..8544)	
Atlantic cod	*Gadus morhua*	tshrb	genomic	full-length	scaffold_2685	join(421..578,724..795,1044..1118,29769..29843,30078..30152,30248..30325,30447..30515,30623..30700,30903..31091,31297..32383,119031..119171)	
Medaka	*Oryzias latipes*	tshrb	genomic	full-length (11 exons)	NC_019870	join(26944021..26944187,26944257..26944328,26946137..26946211,26947045..26947119,26947206..26947280,26947359..26947436,26948502..26948570,26948822..26948899,26949925..26950113,26953071..26954103,26954179..26954373)	
Amazon molly	*Poecilia formosa*	tshrb	genomic	full-length (11 exons)			XP_007572802

Southern platyfish	*Xiphophorus maculatus*	tshrb	genomic	full-length (11 exons)	AGAJ01031179	complement(join(7766..7989,8655..9705,10809..10997,12036..12113,12375..12443,13179..13256,13358..13432,13881..13955,14236..14310,15341..15412,15489..15652))	
Senegalese sole	*Solea senegalensis*	tshrb	genomic	truncated			CBK38914
Stickleback	*Gasterosteus aculeatus*	tshrb	genomic	full-length (11 exons)	AANH01009258	join(72842..73002,73094..73165,74339..74413,77213..77287,77433..77507,77604..77681,78956..79024,79257..79334,79536..79724,81654..82740,82812..83018)	
Bicolor damselfish	*Stegastes partitus*	tshrb	genomic		XP_008299987		
Nile tilapia	*Oreochromis niloticus*	tshrb	genomic	full-length (11 exons)	NT_168107 (LOC100700258)	join(1158..1321,1400..1471,8874..8948,9690..9764,9881..9955,10048..10125,10980..11048,11520..11597,11789..11977,13464..14547,14628..14801)	XP_003460227
Burton's mouthbrooder	*Haplochromis burtoni*	tshrb	genomic				XP_005943102
**Other Gphr genes**							
Sea lamprey	*Petromyzon marinus*	GphRI	mRNA	full-length			AAW80618
Sea lamprey	*Petromyzon marinus*	GpHRII	mRNA	full-length			AAW80619
Japanese eel	*Anguilla japonica*	fshr	genomic / mRNA	full-length (10 exons)	gb|KI305957.1|	join(65335..65492,70883..70954,73337..73411,74363..74437,75890..75964,76524..76604,78585..78653,79719..79793,80511..80696,81460..82576)	BAF79914
Japanese eel	*Anguilla japonica*	lhr	genomic/ mRNA	full-length (11 exons)	gb|KI305181.1|	join(5864..6045,11013..11084,12883..12957,13593..13667,14196..14270,14862..14939,17372..17440,17573..17647,18352..18423,19252..19347,21021..21089,21552..22716)	ACF35638
