# Supplementary material for: Multiple Thyrotropin β-Subunit and Thyrotropin Receptor-Related Genes Arose during Vertebrate Evolution
Source: PLoS One. 2014 Nov 11;9(11):e111361. doi: 10.1371/journal.pone.0111361 (PMC4227674; doi:10.1371/journal.pone.0111361)
Supplement: Table S3 — European eel primer sets for quantitative real-time PCR. (PDF) [file pone.0111361.s007.pdf]

Supplementary table S3: European eel primer sets for quantitative real-time PCR

Genes	Nucleotide sequences	
	Forward 5′ – 3′	Reverse 5′ – 3′
Tshβ	ACTTCTGCGTGGCCATCAAC	GGACTACCAGGCGCTTCACC
Tshβ3	CCCAAAACTACACGCTCTACG	GCCCTTCAACCCACACTTC
Tshra	CACCTACCCCAGCCACTGTT	GGTCGTGCAATACGGTGAAG
Tshrb	CCGGCGTAATGAATGGAC	CTGGCGGTAGTTTCTTCAGG
