# Supplementary material for: Multiple Thyrotropin β-Subunit and Thyrotropin Receptor-Related Genes Arose during Vertebrate Evolution
Source: PLoS One. 2014 Nov 11;9(11):e111361. doi: 10.1371/journal.pone.0111361 (PMC4227674; doi:10.1371/journal.pone.0111361)
Supplement: Table S4 — Database references for the genes in TSHβ and TSHR genomic regions. (PDF) [file pone.0111361.s008.pdf]

Table S4: Database references for the genes in *Tshβ* and *Tshr* genomic region.

	Human chr1	Coelacanth JH126587	Spotted gar LG3	Elephant shark scaffold_89
TRIM33	ENSG00000197323	ENSLACG00000018328	ENSLOCG00000010479	(scaffold_249/305)
NRAS	ENSG00000213281	ENSLACG00000018428	ENSLOCG00000010440	(scaffold_295)
SIKE1	ENSG00000052723	ENSLACG00000018474	ENSLOCG00000010407	SINCAMG00000007993
SYCP1	ENSG00000198765	ENSLACG00000022496	ENSLOCG00000010662	SINCAMG00000007983
SLC5A8l	not found	ENSLACG00000018539	ENSLOCG00000010641	SINCAMG00000007979
TSPAN2	ENSG00000134198	ENSLACG00000018561	ENSLOCG00000010592	(scaffold_33)
NGF	ENSG00000134259	ENSLACG00000018594	ENSLOCG00000017923	(scaffold_365)
**TSHb**	ENSG00000134200	EBI: LK392305	ENSLOCG00000010628	SINCAMG00000007977
KCNA10	ENSG00000143105	ENSLACG00000001741	ENSLOCG00000018067	(scaffold_33)

	Human chr12	Coelacanth JH127380	Spotted gar LG8	Elephant shark scaffold_39
CHPT1	ENSG00000111666	ENSLACG00000000791	ENSLOCG00000015186	SINCAMG00000014969
MYBPC1	ENSG00000196091	ENSLACG00000003430	ENSLOCG00000015188	SINCAMG00000014981
SPIC	ENSG00000166211	ENSLACG00000007445	ENSLOCG00000015193	SINCAMG00000015057
ARL1	ENSG00000120805	ENSLACG00000009274	ENSLOCG00000015193	SINCAMG00000015061
**TSHb2**	not found	EBI: LK392306	not found	SINCAMG00000015310
UTP20	ENSG00000120800	ENSLACG00000010098	ENSLOCG00000015197	SINCAMG00000015283
SLC5A8	ENSG00000256870	ENSLACG00000011968	ENSLOCG00000015201	SINCAMG00000015255
ANO4	ENSG00000151572	ENSLACG00000012620	ENSLOCG00000015202	SINCAMG00000015241

	Zebrafish chr6	Medaka chr5	Stickleback LG XVII	Tetraodon chr11
SLC5A8l	ENSDARG00000003697	ENSORLG00000006499	ENSGACG00000005284	ENSTNIG00000018283
SYCP1	ENSDARG00000003904	ENSORLG00000006520	ENSGACG00000005335	ENSTNIG00000018282
**TSHb**	ENSDARG00000033726	NCBI: LOC101171658	ENSGACG00000005276	ENSTNIG00000018284
TSPAN2-1	ENSDARG00000003754	ENSORLG00000006458	ENSGACG00000005236	ENSTNIG00000018286
NGF	ENSDARG00000014050	ENSORLG00000006424	ENSGACG00000005234	ENSTNIG00000018287
KCNA10-1	ENSDARG00000059935	ENSORLG00000006413	ENSGACG00000005218	ENSTNIG00000018291

	Zebrafish chr23	Medaka chr7	Stickleback LG XII	Tetraodon chr9
**TSHb3**	NCBI: LOC100001596	EBI: LK392307	ENSGACG00000009897	not found
TSPAN2-2	ENSDARG00000059202	not found	ENSGACG00000009844	ENSTNIG00000015021
NTF7	ENSDARG00000058961	ENSORLG00000008499	ENSGACG00000009843	ENSTNIG00000015022
KCNA10-2	ENSDARG00000076854	ENSORLG00000008479	ENSGACG00000009836	ENSTNIG00000015025
LGR6	ENSDARG00000018648	ENSORLG00000008434	ENSGACG00000009806	ENSTNIG00000015029
ETV7	ENSDARG00000089434	ENSORLG00000008468	ENSGACG00000009831	ENSTNIG00000000806

	Human chr14	Coelacanth JH12669	Spotted gar LG7	Elephant shark scaffold_153
SPTLC2	ENSG00000100596	ENSLACG00000014034	ENSLOCG00000008789	NCBI: JH126618.1
NRXN3	ENSG00000021645	ENSLACG00000017041	ENSLOCG00000008835	NCBI: XP_007904238
DIO2	ENSG00000211448	ENSLACG00000016739	ENSLOCG00000008895	NCBI: XP_007904198
**TSHR**	ENSG00000165409	ENSLACG00000016507	ENSLOCG00000008909	NCBI: XP_007904236
GTF2A1	ENSG00000165417	ENSLACG00000016322	ENSLOCG00000008922	NCBI: XP_007904197
STON2	ENSG00000140022	ENSLACG00000016257	ENSLOCG00000008954	NCBI: XP_007904192
FOXN3a (CHES1)	ENSG00000053254	ENSLACG00000015298	ENSLOCG00000008977	NCBI: XP_007904187
KCNK13b	ENSG00000152315	ENSLACG00000014316	ENSLOCG00000009008	(scaffold_645)
CALM1b	ENSG00000198668	ENSLACG00000013233	ENSLOCG00000009055	(scaffold_289)

	Medaka chr24	Zebrafish chr20	Stickleback gr18
SPTLC2b	ENSORLG00000010589	ENSDARG00000074287	ENSGACG00000005382
NRXN3b	ENSORLG00000010661	ENSDARG00000062693	ENSGACG00000005280
DIO2b	not found	not found	ENSGACG00000005269
**TSHRa**	ENSORLG00000010696	ENSDARG00000037195	ENSGACG00000005233
GTF2A1	ENSORLG00000010721	ENSDARG00000011000	ENSGACG00000005224
FOXN3a (CHES1)	ENSORLG00000010729	ENSDARG00000043553	not found
KCNK13b	ENSORLG00000010736	ENSDARG00000043557	ENSGACG00000005206
CALM1b	NCBI: 100049149	ENSDARG00000034187	ENSGACG00000005112

	zebrafish chr17	Medaka Chr22
SPTLC2a	ENSDARG00000018976	ENSORLG00000017650
NRXN3a	ENSDARG00000043746	ENSORLG00000010813
DIO2a	ENSDARG00000094857	ENSORLG00000010816
STON2	ENSDARG00000057452	not found
FOXN3b	ENSDARG00000012833	not found
KCNK13a	ENSDARG00000008212	not found
CALM1a	ENSDARG00000021811	ENSORLG00000017724

	Medaka chr12	Zebrafish chr10	Stickleback gr14
MEGF10	ENSORLG00000014709	ENSDARG00000017229	ENSGACG00000018673
REEP5	ENSORLG00000014495	ENSDARG00000091537	ENSGACG00000018641
CHD1	ENSORLG00000014570	ENSDARG00000014878	ENSGACG00000018644
PAM	not found	ENSDARG00000042071	ENSGACG00000018375
NRG1	not found	ENSDARG00000068458	ENSGACG00000018632
**TSHRb**	ENSORLG00000014222	not found	ENSGACG00000018635
SYK	ENSORLG00000014520	ENSDARG00000008186	ENSGACG00000018650
SPTLC1	ENSORLG00000014633	ENSDARG00000042995	ENSGACG00000018667

References are retrieved from Ensembl and NCBI or EBI when specified. Sequences characterized in the the present study are highlighted in yellow.
